# Implementation of Breastfeeding Policies at Workplace in Mexico: Analysis of Context Using a Realist Approach

**DOI:** 10.3390/ijerph19042315

**Published:** 2022-02-17

**Authors:** Sonia Hernández-Cordero, Mireya Vilar-Compte, Kathrin Litwan, Vania Lara-Mejía, Natalia Rovelo-Velázquez, Mónica Ancira-Moreno, Matthias Sachse-Aguilera, Fernanda Cobo-Armijo

**Affiliations:** 1 Research Center for Equitable Development EQUIDE, Universidad Iberoamericana, Prolongación Paseo de la Reforma 880, Lomas de Santa Fe, Mexico City 01219, Mexico; sonia.hernandez@ibero.mx (S.H.-C.); v.lara.m.16@gmail.com (V.L.-M.); natalia.rovelo@gmail.com (N.R.-V.); 2 Department of Public Health, Montclair State University, 1 Normal Avenue, Montclair, NJ 07043, USA; 3 Department of Social and Behavioral Sciences, Yale School of Public Health, Yale University, 47 College Street, New Haven, CT 06510, USA; kathrin.litwan@yale.edu; 4 Department of Health, Universidad Iberoamericana, Prolongación Paseo de la Reforma 880, Lomas de Santa Fe, Mexico City 01219, Mexico; monica.ancira@ibero.mx; 5 United Nations International Children’s Emergency Fund (UNICEF), Mexico City 11000, Mexico; msachse@unicef.org (M.S.-A.); mcobo@unicef.org (F.C.-A.)

**Keywords:** breastfeeding, working mothers, lactation/breastfeeding room, breastfeeding policies, workplace interventions, breastfeeding-friendly environment, breastfeeding/lactation support, work, worksite, workplace

## Abstract

Return to work is one of the most significant barriers to breastfeeding (BF). Family-friendly policies are critical to ensure that BF and maternal work are not mutually exclusive. This study aims to determine contextual factors and underlying mechanisms influencing the implementation of workplace policies in Mexico. Following a qualitative approach, the study was conducted in the following four cities in Mexico: Mérida, Chihuahua, Guadalajara, and Monterrey. Interviews were conducted in 14 workplaces, and included 49 (potential) beneficiaries, 41 male employees, and 21 managers and human resources personnel. The information collected was analyzed through a deductive thematic analysis and mapped against the Context-Mechanism-Outcome framework of Breastfeeding Interventions at the Workplace. Contextual factors influencing a BF-friendly environment in the workplace were as follows: work-schedule flexibility, provision of lactation services (i.e., BF counseling) other than a lactation room, women’s previous experience with BF and family-friendly environments in the workplace. The underlying mechanisms enabling/impeding a BF-friendly environment at the workplace were as follows: awareness of Mexican maternity protection legislation, usage of BF interventions in the workplace, culture, supervisor/co-worker support and BF-friendly physical space. To achieve a BF-friendly environment in the workplace, actions at the level of public policy and workplaces must accompany adherence to Mexican legislation.

## 1. Introduction

Improving nutrition globally is a matter of social justice and public health. The promotion, protection, and support of breastfeeding (BF) is a proven cost-effective intervention and one of the most promising alternatives to combat malnutrition throughout life, as well as to prevent it from continuing through the intergenerational cycle of families, communities, and nations [1]. Knowledge of all the health and economic benefits that BF brings to children, mothers, families, and society at large, calls for the protection, promotion, and support of BF to be seen as fundamental to equity and social justice [2]. The World Health Organization (WHO) recommends early BF initiation (within the first 60 min postpartum), exclusive breastfeeding (EBF) for 6 months and continuation of BF until at least 2 years of age once nutritionally adequate and safe complementary foods are introduced, starting at 6 months of age [3]. Nearly all women are biologically capable of BF, and very few infants with severely limiting medical disorders cannot be breastfed and/or receive breastmilk [4]; however, BF practices are affected by a wide range of historical, socioeconomic, cultural, and individual factors, where women still face commercial, economic, and social barriers in exercising their right to breastfeed their infants as long as they want to [5]. Thus, BF is a social commitment that goes beyond the mother–child dyad.

Globally, BF practices are suboptimal around the world, by 2019, the rate of EBF was 48.6% in infants aged <6 months and almost 25% of children younger than 12 months were formula fed [6]. In Mexico, in 2018, less than 50% of newborns were put to the breast within 60 min postpartum, the rate of EBF in infants aged <6 months was 28.6%, and only 46.9% and 36.9% continued BF at 1 and 2 years, respectively, whereas almost 43% of children younger than 12 months were formula fed [7]. BF practice indicators are even more discouraging among working women, such as EBF (10.8% in working women vs. 15.6% in non-working women) and continued BF at 2 years of age (8.5% in working women vs. 16.8% in non-working mothers) [8].

The return to work is one of the greatest barriers to BF [5]. Family-friendly policies are critical to ensure that BF and work are not mutually exclusive; however, workplaces often fail to protect, promote, and support BF due to inadequate maternity leaves, flexibility in scheduling to accommodate BF or appropriate breaks and spaces for milk expression and storage after mothers return to work [9]. In Mexico, women working in the formal (work with social security benefits) or informal (self-employment or non-wage contractual arrangement work without access to social protection, e.g., maternity leave benefits) sector are 20% less likely to ever breastfeed (children < 24 months), 10% less likely to have an early onset of BF (during the first hour of life) and 20% less likely to EBF compared to unemployed women [10]. This is a worrisome situation as working mothers should not have to choose between employment and BF, as both are key for economic and social development; and sometimes women’s employment is the only source of family income. Policies and strategies to promote and support BF in the workplace not only contribute to nutrition and health outcomes for children and mothers, but are also beneficial for companies, as they have been reported to reduce absenteeism and health care costs, and improve employee retention, productivity, loyalty, and morale [11]. In addition, BF policies are important for promoting gender equality both in the labor market and in the domestic sphere. Including fathers in leave policies can promote a fairer distribution of infant care between men and women (parental co-responsibility), and hence contribute to gender equality in the country. At the same time, BF breaks and lactation rooms allow women to exercise their rights to work as well as to breastfeed [12]. Finally, policies to support BF at the workplace, along with maternity, paternity, and parental leave, are essential for a comprehensive social protection system and employment strategies [13].

In Mexico, the current legislation for maternity protection is included in the Mexican Constitution, which provides maternity leave as a pre- and post-natal leave for a total of 12 weeks (six weeks for each period), guaranteeing the mother’s full salary, as well as the preservation of her employment and its legal benefits [14]. It is only during the first six months after returning to work, following the end of maternity leave, that working mothers are entitled to two extra breaks per day of 30 min each, for BF or for the manual extraction of milk at a designated place, or a reduction of one hour in the workday [15]. There is no research in Mexico studying policies at the workplace to promote, protect and support BF. The aim of this study was to determine contextual factors and underlying mechanisms influencing the implementation of BF policies at the workplace in Mexico. We did so by conducting an analysis that was inspired by a realist evaluation approach using a Context-Mechanism-Outcome (CMO) framework that was developed in a prior realist review [16]. A realist evaluation is a realist research approach using primary data to answer one or all the questions of ‘what works, how, why, for whom, to what extent and in what circumstances, in what respect and over what duration?’ [17]. At minimum, realist evaluations answer the ‘how?’ and ‘why?’ questions.

## 2. Materials and Methods

### 2.1. Design

In order to achieve the aim of the study, a qualitative approach was adopted. In a cross-sectional study, data were collected through semi-structured interviews. Compared to structured interviews, this method has proved successful in allowing reciprocity between interviewer and participant, enabling the interviewer to improvise follow-up questions based on the participant’s answers, and providing space for participants’ individual verbal expressions [18,19].

### 2.2. Participants and Settings

Four cities were selected to perform the analysis. The four cities were purposely selected based on a Ministry of Labor dataset on public and private workplaces. The cities included were Mérida (Yucatán State), Chihuahua (Chihuahua State), Guadalajara (Jalisco State) and Monterrey (Nuevo León State). Two of them from the north (Nuevo León and Chihuahua), one from the South (Yucatán) and one from the Center (Jalisco) of the country. In addition to their locations, these cities were selected because of existing work relations with several local institutions that could eventually facilitate the research team to contact different worksites for the gathering of information. For the eligibility of workplaces within the selected cities, the criteria were: (i) to be included in the databases provided by the Ministry of Labor in 2019; (ii) to have complete information in the database (including an email address for sending the questionnaire); and (iii) to have more than 50 employees registered on its payroll. Workplaces that lacked information on the number of employees were excluded. The rationale for including workplaces with a minimum of 50 employees was to ensure that workplaces complied with the above-mentioned Statement by the Labor and Management Sectors for the Promotion of Maternity Protection and the Promotion of Breastfeeding in the Workplace agreement [20]. The workplaces (*n* = 58) were contacted through an invitation letter addressed to the managers or human resources, and if they accepted, they facilitated the contact with potential participants (*n* = 13). Interviewees were classified as follows: (i) beneficiaries and potential beneficiaries (female employees); (ii) male employees, and (iii) managers and human resources personnel. The participants were recruited through convenience sampling.

### 2.3. Ethical Considerations

Before beginning data collection, the research protocol was approved by the Ethics Committee of the *Universidad Iberoamericana* in Mexico City and the necessary procedures were carried out with the authorities of each workplace to obtain permission to conduct the study. The safeguarding of ethical principles regarding the participation of individuals was formalized through the development of a consent form (Supplementary Material S1), which included a description of the study and the implications of participation, using clear and accessible language. We were able to clarify any doubts via contact with the investigator. To guarantee the confidentiality and anonymity of the information, each interviewee was identified with an ID (City_Workplace_Type and number of interviewee) that replaced her or his name in the interview transcript. This research adhered to the ethical principles for research of the Belmont Report, which are: respect for persons, beneficence, and justice [21]. They were guaranteed by providing the possibility of dropping out without any justification or disadvantage for the participant, and because the interviewees’ participation does not imply any risk for their person.

### 2.4. Data Collection

The original protocol included on-site visits to workplaces; however, due to the COVID-19 pandemic, only workplaces in Chihuahua and Mérida were visited. Information from the remaining cities (Guadalajara and Monterrey) was collected through phone calls or video calls via the Zoom platform. Data were collected between February and November 2020. The semi-structured interview guidelines were developed based on a literature review, and there was a specific guideline for each type of actor (Supplementary Material S2). Consent was granted verbally to record each of the interviews. A total of 111 interviews were conducted in 14 work centers and distributed as follows: 49 beneficiaries or potential beneficiaries, 41 male employees, and 21 managers and human resources personnel (Table 1 and Table 2).

### 2.5. Qualitative Thematic Analysis

The information collected was analyzed through a deductive thematic analysis and mapped against the CMO Framework of Breastfeeding Interventions at the Workplace to understand the context and the main facilitators and barriers influencing the [16] implementation of BF policies in the workplace in Mexico. The pragmatic CMO framework was considered adequate since it was developed with the purpose of identifying contextual factors influencing the acceptance of BF workplace interventions.

The framework identified the following three mechanisms that must be activated for an effective intervention: (i) awareness of workers, supervisors, and co-workers about the availability of the right to a given intervention; (ii) changes in workplace culture, supervisor and co-worker support, and appropriate physical environments, and (iii) provision of time to breastfeed or express breastmilk during work hours. The CMO Framework of Breastfeeding Interventions at the Workplace describes that BF workplace interventions always act through the contextual factors in which the interventions take place, such as the presence of shift work or maternal education level. Such contextual factors can influence the way the interventions work. For example, in an environment where the working mothers work in shifts, the sole provision of a BF intervention may not be sufficient and needs the activation of the “Sub-outcome and mechanism” of supportive co-workers and supervisors [16]. Besides the three previously described mechanisms that need to be activated for an effective BF workplace intervention, the CMO Framework of Breastfeeding Interventions at the Workplace also identified various “Sub-outcomes and Mechanisms” [16]. This category in the CMO Framework describes an outcome of the intervention that is not the main target outcome (the change in BF habits) but results from the intervention and is needed so that the intervention can be fully effective. For example, supportive co-workers can take care of immediate tasks that need to be covered while the BF colleague takes a break for lactation. This behavior allows the working mother to use the lactation break, and thus, indirectly provides the needed time to breastfeed.

### 2.6. Data Analysis

After data collection, the audio recordings were transcribed *verbatim* using the Word Processing Program. Two researchers (VLM and NRV) coded the interviews in *Dedoose* (version 9.0.17) (SocioCultural Research Consultants, Manhattan Beach, CA, USA), using the codebook developed for this study (Supplementary Material S3). The categories and subcategories were based on the following CMO Framework components: (i) Context, (ii) Mechanism, and (iii) Mechanism and Sub-Outcome [16]. However, in addition to these deductive codes, other inductive codes that emerged during the interviews were added (e.g., previous BF experiences and professional or peer support for BF). Discrepancies in coding were resolved through discussion with a third researcher (SHC).

## 3. Results

Fourteen workplaces from four cities, settled in four different Mexican states participated in the study, including Chihuahua (*n* = 6), Mérida (*n* = 4), Guadalajara (*n* = 3) and Monterrey (*n* = 1). The workplaces studied were mostly from the private sector (*n* = 11) with different lines of business, such as commerce (*n* = 4), education (*n* = 2), technology (*n* = 2), communication (*n* = 2), health (*n* = 2), environment (*n* = 1) and tourism services (*n* = 1). The included workplaces had an average of 327 employees, with more male than female personnel. Only five of the studied workplaces had a lactation room at the time of data collection (Table 1).

The study population included beneficiaries or potential beneficiaries (44.2%), male employees (36.9%), and managers and human resources personnel (18.9%) with an average age of 36.6 years old. Most interviewees were married or in free union (83.8%) and held a bachelor’s degree (60.4%). The most prevalent type of interviewee was the beneficiaries or potential beneficiaries of BF policies in the workplace (Table 2 and Supplementary Material S4).

**Figure 1:**
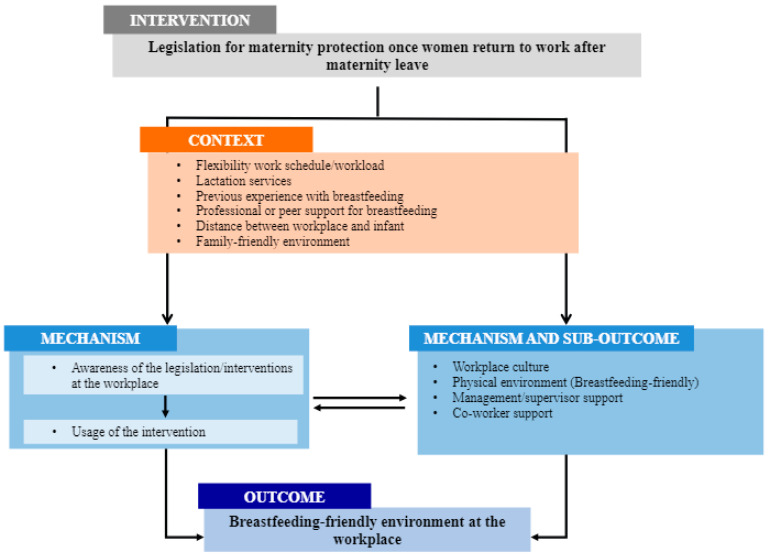
Factors contributing to a Breastfeeding-friendly environment at the workplace in Mexico. Based on the CMO Framework of Breastfeeding Interventions at the Workplace. CMO: Context-Mechanism-Outcome.

We focused our thematic analysis on the contexts, mechanisms, and outcomes to identify reasons behind promoting a BF-friendly environment at the workplace, for women who want to continue BF once they return to work after maternity leave, described on Figure 1, adapted from Litwan and colleagues [16].

### 3.1. Context

#### 3.1.1. Flexibility Work Schedule/Workload

Mexican legislation states that women are entitled to two extra breaks per day of 30 min for BF or breastmilk manual extraction at a designated place, or a reduction of one hour in the workday, for a period of only six months [15]. This gives some flexibility in women’s work schedule. However, even though it is stipulated by law, many women do not *de facto* have or use such breaks or hour reduction, either because of the type of work, supervisor’s inflexibility or the mother’s own preference or unawareness.

*[Schedule flexibility] “… Yes, they gave me a time to choose, you know that the law says that it is a half hour to breastfeed, they gave me the time to choose whether to come in late or leave early, because here you can’t take your half hour to go and breastfeed your child, nor is there a space for them to bring the baby to you. I know there are places that have a lactation room, and you can go to express milk during those half hours. So, I would leave early, however, I can tell you that there were days that they would not let me leave at my time. The work that our area has sometimes did not allow it…”*

(Beneficiaries or potential beneficiaries, Chihuahua, [B02])

*[No schedule flexibility] “… No, it was normal, I didn’t have that thing about leaving earlier or coming in later. It was a normal schedule…”*

(Beneficiaries or potential beneficiaries, Merida, [B03])

Importantly, there were some cases in which workplaces were perceived to be very willing and flexible in allowing women to manage their schedules based on their needs, such as providing the option of arriving an hour late, having two hours of lunch or leaving an hour earlier. Likewise, some cases, in addition to what is stipulated by law, provide the flexibility for women to use the lactation room as often and as long as necessary, seeking to identify the best options to comply with legislation without affecting their productivity.

*[Schedule flexibility] “… Working mothers can enjoy the reduction of their working day for breastfeeding period once their maternity leave is over […] through a request they make directly to their immediate supervisor where they present the schedules that best suit them for the attendance of their child, […] and she explains to us if she wants to arrive an hour later than when she starts work, if she wants to take two hours for lunch or if she prefers to leave an hour earlier than her working time, as she decides, to breastfeed her child. In addition, as many times as she requires during her work day in the space we have designated for breast milk expression […] she can use them as long and as often as she needs to.…”*

(Manager and HR personnel, Guadalajara, [RRHH02])

#### 3.1.2. Lactation Services

The workplaces studied, with a few exceptions, did not implement any measures, infrastructure, or activities beyond what is required by Mexican law, and most of them did not even have a lactation room. In those workplaces that reported additional services and support, they reported having follow-up checks for pregnant and lactating women, some health promotion covering maternal and child health topics, talks about BF focusing on its benefits, recommendations on duration, or the availability of materials on the subject.

*“… Once they come off maternity leave, we are aware of when they return because at that time we contact them and talk to them about breastfeeding, we give them a talk on breastfeeding, benefits for them, for the baby, etc., and we provide follow-up, we explain to them what the breastfeeding period is and after that period, if they need more days, how they should request it, because they can request more days than the period that is normally given in the workplace. What else? She is monitored every month to see if there is no problem during lactation, if so, she is referred…”*

(Manager and HR personnel, Mérida, [RRHH])

*“…Mmm yes, I tell you, the flexible schedule, and also many people come to talk to us about everything related to health, maternity and baby…”*

(Male employees, Chihuahua, [H02])

#### 3.1.3. Previous Experience with BF and Professional or Peer Support for BF

Most of the participating women had problems establishing and continuing BF prior to their return to the workplace, and did not have professional or peer BF support, nor advice on how to manage BF when returning to work. Some women mentioned the time-demanding jobs, where it is not feasible to take breaks to extract milk, which makes it difficult to continue this practice once returning to work.

*[No support] “…Yes, he was hungry, well, you could see it. Even when I extracted my milk, I had very little. Besides, with the cesarean section it was very tiring to breastfeed in one position and I could not tolerate the pain in my back, and she did not want to get off the breast, so I decided to help myself with formula from the beginning…”*

(Beneficiaries or potential beneficiaries, Chihuahua, [B01])

*[No support] “…Yes, in fact, at the beginning it was only breast milk and then when I was getting ready to go back to work, I had to combine formula with breastfeeding, that’s how it was for both children…”*

(Beneficiaries or potential beneficiaries, Chihuahua, [B03])

Among some women, an aspect that mediated these difficulties was a prior positive BF experience.

*[Success with BF] “… The first one, the truth is, with the first one I didn’t work, and I was at home, and I was able to give him milk for almost two years. With this baby, he is exactly one year and two months old, I continue to breastfeed him when I come home from work and then at night, he continues to drink something…”*

(Beneficiaries or potential beneficiaries, Mérida, [B01])

*[Success with BF] “…My mom, with my first baby. With my first baby I learned many things, my mom showed me, she bought me the breast pump, I mean, a woman’s body is wonderful because I didn’t even have to fight to breastfeed, I always had enough milk, always, always, always, to this day. So, I was learning by myself and with, well, my mother’s workplace. And now that my second baby was born, well, I was released on September 29, by October 2 I was already doing my milk bank and, practically, you learn as you go along…”*

(Beneficiaries or potential beneficiaries, Chihuahua, [B04])

#### 3.1.4. Distance between Workplace and Infant

Working mothers mentioned the distance between the workplace and their young children as a barrier to continue BF when returning to work. On the other hand, a short distance acted as a BF facilitator.

*[Enabling factor] “… I took the breastfeeding hour half an hour before going to work and I left half an hour before my work schedule. I also have two hours for lunch, I live very close so I would go home, that also helped me…”*

(Beneficiaries or potential beneficiaries, Mérida, [B01])

*[Barrier] “… However, now an hour or two away, because she’s not going to breastfeed the boy or the girl, she’s not going to be able to go home [in such a short time] …”*

(HR personnel, Guadalajara [RRHH01]

There is the perception that having a daycare center within the workplace or having the flexibility of bringing the baby to mother’s workplace, so she can breastfeed, would facilitate women to continue this practice once they return to work.

*[Enabling] “… So, yes, it should be the right to ask for a lactation room so that you can do it, but if we go to those points, we as a clinic would ask for a lactation room or a daycare because I think the daycare would be easier […] you being here working, “you know what? I feel that my breasts are full” that the clinic can provide us, what would it be? half an hour or an hour to be able to go (to the daycare), give milk to the baby and come back […] They give you your baby, you are in contact with him, seeing that he is well, you give him his two feedings. You are happy, you are not uncomfortable, the baby is full, and you go on with your work. That’s what it should really be, right? but well… it’s fair to dream…”*

(Beneficiaries or potential beneficiaries, Mérida, [B04])

*[Enabling] “… it means that the workplace has to help you as a woman to continue breastfeeding […] give them an hour so that they can go out, or provide them a lactation room and a refrigerator so that they can extract their milk […] because it would be strange in the bathroom, wouldn’t it? […] it would be weird, wouldn’t it? even for hygiene reasons. Now there are workplaces that even have a daycare downstairs, you can go up and down. So, yes, it is more like trying to invite workplaces to take more care of their women, and of the fathers too, because there are fathers who are very supportive of breastfeeding at work…”*

(Beneficiaries or potential beneficiaries, Chihuahua, [B07])

### 3.2. Mechanism

#### 3.2.1. Awareness about Maternity Protection Legislations and Interventions Promoting and Protecting BF in the Workplace

Even though working women and men were aware of the existence of the legislation, some of them did not know what they are entitled to by law, nor what was being done at the workplace to guarantee their rights and to protect and support BF.

*[Unawareness of workplace policies] “… Well, I don’t see…I haven’t seen in my department that they tell them anything, on the contrary, they give them support in that sense, that, for example, they work a period of eight hours, right, the working day, and they give them the possibility of leaving an hour earlier to be able to exercise it…”*

(Male employees, Merida, [H01])

*[Unawareness of workplace policies] “… (was asked if she had read or been told that it is a woman’s right to have breastfeeding protected and supported in the workplace) I didn’t know that [laughs], I hadn’t heard anything…”*

(Beneficiaries or potential beneficiaries, Chihuahua, [B01])

*[Unawareness of workplace policies] “… Well, not directly by the workplace, as I said before […] it is possible for women to take some time to extract their milk, but I don’t know the workplace policy…”*

(Male employees, Chihuahua, [H03])

Important to notice is that some workplaces make an effort to promote the benefits as well as the actions they implement to support BF among working women once they return to work.

*[Promotion by workplaces] “… No, in fact, all the staff is kept informed that the lactation room has been relocated, and not only for the internal staff or for the internal clients who are our employees, but also in case it is necessary for the guests, which has already happened once a couple of years ago. It is important that they are aware so that if someone asks them, they have the information to be able to say: “yes, we have a lactation room in the workplace, you can use it”, that’s why it is constantly maintained…”*

(Beneficiaries or potential beneficiaries, Guadalajara, [B02])

#### 3.2.2. Usage of the Intervention

When there is support for the use of the lactation room or other benefits (both, those by law and additional ones), these are used and appreciated. Women have different preferences as to how to use or exercise their maternity rights. Some prefer to change their schedule (either arriving later or leaving earlier), while others prefer to use BF breaks for milk extraction. In this sense, supervisors usually have no interference and allow mothers to decide.

*[Usage of intervention] “… I would think that about five women have used the lactation room more or less. This year there were literally about five, but because of COVID it has not been used […] I had a nutritionist in my team and this year she got relief from her baby and was in quarantine, she wasn’t able to use the lactation room either, the one who was able to use it was a previous employee I had and she did use it […] However, yes, if they are given the opportunity to have their breastfeeding times or have a flexible schedule, they try to use the lactation room…”*

(Manager and HR personnel, Monterrey, [RRHH01])

*[Usage of intervention] “… Yes, of course they can use it without any problem, remember that they also have their half hours […] in the end it is their decision, one girl was out because she was considered vulnerable because she was breastfeeding, so when she returned she had two months left and the agreement was that she should leave an hour earlier, but, if she decides, she can go and use the lactation room without any problem, in fact, there is no one who questions this issue, there is only a record…”*

(Manager and HR personnel, Monterrey, [RRHH02]

### 3.3. Mechanism and Sub-Outcome

#### 3.3.1. BF Culture in the Workplace (Workplace Culture)

There is a general awareness of maternity protection legislation; however, BF is considered a personal matter, thus the workplace is not identified as a space to support it. In addition, there is a perception on the part of both female and male workers, as well as managers, that the number of potential users of the lactation room is limited (fluctuating between one or two per year), so the space would be unnecessary.

*[Perception that the number of potential users is limited] “… Well, I don’t know how… no, I don’t know. I mean, I feel that time has passed for me, I was fine, I don’t have a complaint, so, I don’t think there are that many of us who have children, so, if the question is whether I think it is necessary, I really don’t. Outside, for example, a clothing manufacturing plant, where most of them are women, maybe yes…”*

(Beneficiaries or potential beneficiaries, Chihuahua, [B03])

*[Perception that the number of potential users is limited] “… (was asked if a lactation room is necessary) Well, if our maternity rate were higher, I think it would be necessary, it would definitely be a yes, we would be thinking about having a lactation room, but our rate is so low that it would be a facility, an useless space that we wouldn’t use…”*

(Manager and HR personnel, Chihuahua, [RRHH01])

*[(lack) Incentives enabling BF-friendly environment in the workplace] “… We have talked to managers, they tell you that the initiative is very cool, but they don’t call you back; when you go back to “bother” them, so to speak, one of the things they tell you is that “ups!, I have to submit this project for approval because it costs, if it were a project that did not cost anything there would be no problem, I would give the green light, but it is an initiative that costs because if I do not have the space, I have to build it and if I have it, I have to enable it”…”*

(Manager and HR personnel, Mérida, [RRHH])

*[(lack) Incentives enabling BF-friendly environment in the workplace] “…our main duty is the academy, we need office spaces for teachers, we need classrooms for our students… a series of priorities have been generated, that is why, it seems to me, that the lactation room is taking shape so far…”*

(Manager and HR personnel, Guadalajara, [RRHH01])

There were some workplaces with a “corporate culture”, where other policies and actions aimed to benefit its employees were in place. Although their interventions and actions were not focused on BF per se, they create a more family-friendly environment. All these workplaces offer benefits beyond the law or additional benefits (i.e., an extra hour to leave early or reduction of work schedule to six hours). As some workplaces recognize the increase in the number of working women, they perceive the need to include workplace maternity benefits and the formation of a more family-friendly environment, including a more BF-friendly environment.

*[Importance of a BF-friendly environment in the workplace] “… Well, I think it is very important, because in addition to the fact that mothers do not feel included, in terms of the compatibility of work and family life, I believe that in the sense of belonging to the workplace. It has favored the work environment and moms feel more accepted. The truth is that it was really appreciated both by the mothers and even by male workers. They told us that it was good that we had this space because later they realized that their female colleagues were suffering in the offices, they would go into a meeting room, so they did not have this decent and private space for milk extraction. So, I think that was the main benefit, that they felt confident and the environment among the working mothers was favorable…”*

(Manager and HR personnel, Guadalajara, [RRHH02])

*[Incentives enabling BF-friendly environment in the workplace] “… They let us know that there was another (recognition) called “Family Responsible Company”, and within the guidelines, because we were already giving courses on breastfeeding, we were providing information and training, we were explaining to people the importance of this, but we did not have a space as such to provide support to a person who needed it. So, when we looked into this recognition, we realized that one of the things they asked us to do was to have a lactation room, and we really liked the idea because we had already thought of something like that, but the idea was not quite ready. The Ministry gave us all the guidelines to put it in an orderly manner and give it that formal protocol…”*

(Male employees, Guadalajara, [H01])

Finally, some participants mentioned the importance of mechanisms and strategies to improve the BF-friendly environment, for example, the aim of achieving certification by a higher authority, in this case the Ministry of Labor, and compliance with national standards (specifically, it was mentioned among the interviewees the non-mandatory Mexican Standard NMX-R-025-SCFI-2015 on Labor Equality and Non-Discrimination [22]).

*[Incentives enabling a BF-friendly environment in the workplace] “… No, no, not at all, because it is properly stipulated. In fact, we are certified by the Equality Norm where this certificate supports us, allows us to disseminate, to promote spaces and, above all, to raise awareness among managers that they should provide facilities for working mothers, since this is a right for children, so what we promote is the right to breastfeed their children…”*

(Manager and HR personnel, Guadalajara, [RRHH02])

#### 3.3.2. Physical Environment

Although Mexican law stipulates that there must be a suitable place for milk extraction once mothers return to work after maternity leave, there are workplaces that still do not have such places available, forcing mothers to find inadequate places to do so. Some of the spaces mentioned by the interviewed mothers include toilets, unoccupied boardrooms, or even closets and their own cars. Another challenge faced by women who want to continue BF, once they return to work, is the lack of exclusive storage space for the milk extracted during working hours, so on several occasions they had to throw the milk away, fearing that it would be contaminated by having extracted it in the bathroom, or because they kept it in refrigerators that had other types of food.

*[No physical space] “… I extracted myself here, but since there is no lactation room, I regularly went to the bathroom, but I didn’t keep the milk, I threw it away, it was just to continue producing…”*

(Beneficiaries or potential beneficiaries, Chihuahua, [B01])

*[No physical space] “… Well, there are other foods [in the fridge], the truth is that there were occasions when I kept it [the breastmilk] in there [in the fridge], but I don’t know. I didn’t have the confidence to give it [the breastmilk] to him because the fridge is really public…I usually threw it away [the breastmilk] …”*

(Female employee, Mérida, [B01])

On the other hand, very few studied workplaces already have a lactation room or are in the process one of implementing one. Those workplaces have a more “corporate culture”, aiming to benefit its employees.

*[Available physical space] “… Look, when it was my first delivery, I extracted in the nurses station. In fact, the nurse was there in front. So, it was not very practical because if someone wanted to come in or if there was someone sick and you were in the back extracting milk, wasn’t it? With the second baby there was already a specific space, then there were chairs, there was air conditioning, there were all the conditions, there was a logbook, stereo, you could play music, there was an instruction manual, there was a refrigerator, there was everything. […] So, in the first one, although there was space, it was a bit uncomfortable; in the second one everything was very easy, everything was close at hand, everything was clean, everything was new and, to tell the truth, it was much easier…”*

(Beneficiaries or potential beneficiaries, Guadalajara, [B01])

#### 3.3.3. Management/Supervisor Support

Although there is a perception that women can exercise their right under Mexican law, and some women, male collaborators and supervisors stated so, some of the female participants pointed out that they had to request access to such rights from their supervisors, often referring to current legislation.

*[Inadequate support] “… No, in fact, this subject had not been mentioned to me and I had to mention it to my boss […] because I was coming, I had already returned from my maternity leave, and I had been going out late for a week. I told him “I think that by law I have a provision or a benefit as a worker, I don’t know if it is half an hour or an hour, in different workplaces they work it differently”, and he said “but it is not by law or yes?”, and I said “Yes, I think it is by law”, and I had to make him a copy of the law and I sent it to him by WhatsApp so he could read it and he said “well, you can take the half hour”…”*

(Beneficiaries or potential beneficiaries, Mérida, [B07])

*[Inadequate support] “…I lost my maternity leave because I gave birth early, so it was only a month and then I started to go to work. In my previous job I was supposed to leave early to breastfeed, but it was very complicated, it was not easy because there was always demanding work. The boss was demanding and then there was no place where you could extract your milk and store it. So, no, they didn’t have those conditions…”*

(Beneficiaries or potential beneficiaries, Chihuahua, [B16])

#### 3.3.4. Co-Worker Support

In general, a supportive environment is identified among the collaborators, towards BF mothers and their integration into work activities, considering the benefits they must receive by law. However, despite an awareness of the legislation on maternity protection, there are perceptions of some activities that might be affected when mothers exercise their rights, and possible non-compliance on the part of collaborators.

*[Perceived support] “… since we have become familiar with the fact that it is something normal, we all accept the decision, if a colleague is at a work center, we support her or we can solve the details before she arrives. In the event that she has to leave early, we all try to do the work that will be done in the absence of this person…”*

(Male employees, Merida, [H03])

*[Inadequate support] “… What I have perceived, but we have not surveyed, there are people who are bothered by the fact that women leave earlier because they consider that they are burdened with too much work, but honestly they are a couple of isolated comments, the others do not even mention it, I don’t think they even have it in mind…”*

(Manager and HR personnel, Chihuahua, [RRHH01])

Representative quotes from semi-structured interviews are shown in Table 3 and Supplementary Material S5.

## 4. Discussion

To our knowledge, this is the first study in Mexico describing the state of BF policies in the workplace, by determining the contextual factors and underlying mechanisms that enable or constrain the implementation of strategies to protect, promote and support BF in the workplace. To pursue this objective, a realist evaluation approach was used through a Context-Mechanism-Outcome (CMO) framework [16].

The Mexican legislation, although it would benefit from several modifications [23], aims to promote equality for all working women and the health and safety of the mother and the child, thus acting as an enabling factor for women to continue BF once they return to work. Despite the existence of legislation, many Mexican working women are sometimes forced to demand the observance of their rights, for which they need to be aware of the benefits stipulated by law. The study identified contextual factors contributing to/impeding a BF-friendly environment in the workplace. These factors are the flexibility in the work schedule or workload, the provision of lactation services (i.e., BF counseling) other than a lactation room, women’s previous experience with BF and family-friendly environments at the workplace. In the surveyed workplaces, both extremes were identified, i.e., workplaces where little flexibility in work-schedule was commonplace, few or no BF services were available (including the absence of lactation rooms), or few policies to promote a family-friendly environment were adopted. On the other hand, few of the studied workplaces already have a culture in which the health and well-being of their collaborators is being promoted, including actions enhancing a BF-friendly environment.

We identified some underlying mechanisms and sub-outcomes that promoted a BF-friendly environment at the workplace. As for the contextual factors, we identified, on the one hand, workplaces where neither working women, male collaborators nor the managers and supervisors were aware of the Mexican maternity protection legislation, or the actions implemented in their workplace to support women to continue BF. On the other hand, there are workplaces in which legislation and measures are known and their application and use are promoted among working women and men. Similarly, some of the studied workplaces showed greater support to women to continue with BF once they return to work from the supervisor and/or coworkers and a physical environment and culture that was amenable, as a BF-friendly environment.

Identifying contextual factors and mechanisms to foster BF-friendly environments are of great relevance for issuing recommendations to protect, promote and support BF in the workplace. BF is a socially optimal investment and constitutes a shared responsibility that requires the involvement of all employees and employers to ensure the creation of environments for BF mothers in the workplace. Breastfeeding is currently promoted as a shared responsibility; thus, it is considered as a collective cultural fact that requires an intersectoral and community approach. This is an opportunity to take care of the health of infants and nursing mothers with the support of all actors in society. It is expected that this system will help create a BF-friendly environment and protect BF against industry influence. In Mexico, during the January-March 2021 period, almost 50% of all women in reproductive ages were working [24]. It is socially fair to respect their BF choices even after they return to work. This is also a right strategy from a gender-equity perspective.

To complete the objective of identifying contextual factors and underlying mechanisms influencing the implementation of BF policies at the workplace in Mexico, data were analyzed by using a CMO framework from a realist review [16]. A realist approach assumes that the intervention’s effectiveness is conditional on the context and seeks to understand the key contextual factors and underlying mechanisms responsible for the observed effect of the program [17]. Given the high dependency of BF interventions in the workplace on individual’s response and the wider context in which mothers and families decide to (or not to) breastfeed, a realist approach is the most suitable approach to follow, given its aim to disentangle underlying mechanisms for a given outcome in relation to contextual factors. Following a realist evaluation approach, clear hypotheses could be developed about how, and for whom, the intervention works and for whom the intervention does not work, always referring to the context in which the intervention took place. In addition, a realist approach does not only account for statistical inferences but also for plausibility and adequacy considerations and does, therefore, follow Victora and colleagues who proposed to not solely focus on statistical inferences, but broaden the view about why something works (or does not work) [25]. Moreover, workplace BF interventions are complex service interventions, which can be best evaluated by using a realist approach [26].

By using an already developed CMO framework on effective workplace BF interventions [16], we organized the analysis of data along the concepts developed in this CMO framework. By not only using deductive codes (which were derived from the previously developed CMO framework), but also inductive codes that emerged from the interviews, we were open to identify new concepts (e.g., previous BF experiences) in addition to the concepts identified in the realist review that gave rise to the CMO framework. It is further to notice that the design of the study and the development of the interview guide was independent of the CMO framework. Therefore, the analysis was not only a confirmatory exercise to ensure an existing framework but was rather guided by an existing framework, thus, helping to identify existing relationships more easily.

There are few studies addressing the promotion, protection, and support of BF in the workplace using qualitative methodology. In this case, this is the first study that describes the implementation of BF policies in Mexican workplaces. The qualitative studies identified focused on exploring the perception of the need for interventions to support mothers to continue BF once they return to work after maternity leave [27], as well as barriers faced and facilitators to implement them [28]. The results agree with previous findings from qualitative studies. For example, others have also reported that the worksite is perceived as a place where BF is not discussed. Scott et al. described in a study conducted at health care settings in the United States, that female health care professionals are “concerned that they will be perceived as less productive, if they raise their personal BF needs at the workplace” [28]. On the other hand, Chang et al. identified, in a systematic review of qualitative studies, the lack of a BF culture in study workplaces as well as a poor education and knowledge about BF in several countries around the world (the United States, Malaysia, Iran, Australia, United Kingdom, Ireland, and New Zealand) [27]. Both Chang´s systematic review as well as our study show the role of supervisors and colleagues as an important barrier or facilitator for working mothers to continue BF once they return to work. Finally, consistent with a recent systematic review aiming to describe workplace interventions to promote, protect and support BF practices among working mothers globally (which included quantitative and qualitative studies) [9], the present study identified the poor knowledge on legislation to protect maternal and child health, including BF, among working women, employees, and co-workers. Taken together, all studies highlight the importance of developing written policies at different levels (national, local and within each worksite) as well as educating co-workers, directors, human resources personnel and mothers about the importance of BF and the local maternity protection legislation to promote a BF-friendly environment.

One result to highlight is that workplaces with a corporate culture (workplaces where other policies and actions aimed to benefit its employees were in place) have more benefits for their employees. This study identified that most of the studied workplaces complied with the requirements of the legislation. However, a few of them reached beyond what is required by law, including, for instance, giving an extra hour for breast milk extraction, more flexibility in the working schedule or having information about maternal and child health and nutrition. One of the incentives for going beyond the current legislation was that the workplaces were certified by a higher authority. For instance, the Mexican Official Norm 25 (2015) on Labor Equality and Non-Discrimination is a voluntary certification that helps to recognize workplaces with practices that favor the integral development of workers [22]. To achieve certification, workplaces receive a third-party audit to verify that their policies and practices comply with the requirements, some of which are: incorporating the gender and non-discrimination perspective in recruitment processes; ensuring equal pay; carrying out actions of co-responsibility between work, family, and personal life of their workers, among others. Another example of such incentives, in August 2016, the labor and business sectors of Mexico signed the document, called “*Pronunciamiento de los Sectores Obrero y Patronal para el Fomento de la Protección de la Maternidad y la Promoción de la Lactancia Materna en los Centros de Trabajo*” (Statement by the Labor and Management Sectors for the Promotion of Maternity Protection and the Promotion of Breastfeeding in the Workplace), in which they committed to promote the installation of lactation rooms in workplaces with more than 50 workers, in adequate and hygienic places [29].

Thus, family-friendly policies, including those used to enhance a BF-friendly environment at the workplace are urgently needed to protect women´s and child´s health, promote gender equity and protect women’s participation in the workforce. There is a substantial need to change the culture about BF within workplaces, which requires more than legislation. It is linked to a change in the working culture, including demystifying BF as feminine, and any machoistic view that implies that is incompatible with work and productivity. All of it is consistent with the findings of a recent systematic literature review [9]. Moreover, the Becoming Breastfeeding-Friendly (BBF) Committee in Mexico recommended that Mexico should work towards having better conditions for BF working mothers including a BF-friendly working environment [30].

Although this was not the aim of the study, it is important to highlight the gap in legislation to protect, promote and support BF among women working in the informal sector, a subsection of the population that is completely unprotected in this regard. Additionally, it is important to include paternity leave and its importance in protecting and supporting BF in the political agenda [31,32]. In September 2021, modifications to Article 94, Section VIII were approved, which allows male workers in Mexico to take a paid paternity leave of 15 working days for the birth of their children and likewise for the adoption of an infant. Previously, fathers had a paternity leave of 5 working days [33]. Before this modification, Mexico was behind Colombia, Ecuador, Peru, Uruguay, Paraguay, and Venezuela, all of which offer eight to 14 days of paternity leave with 100% salary [13]. Maternity and paternity leave is important for promoting gender equality both in the workforce and in the domestic sphere by contributing to a fairer distribution of care work between men and women [34].

This study has some limitations. For example, the workplaces included in this study are from the main cities in Mexico and have more than 50 employees registered on its payroll. Although this helped to capture large working sites, it excludes environments such as rural sites (agricultural work sites), or other employment in smaller cities. The rationale for including workplaces with a minimum of 50 employees was to ensure that workplaces had to comply with the above-mentioned Statement by the Labor and Management Sectors for the Promotion of Maternity Protection and the Promotion of Breastfeeding in the Workplace agreement [20]. The study also excluded informal working sites, which is a substantial issue in countries such as Mexico, as 54.5% of the women are employed in the informal sector [24]. Another important limitation was the interference of COVID-19 pandemic during the data-collection stage; the context was not equivalent in pre- and pandemic sites. On the other hand, the use of information technology communication platforms for interviews might have previously been considered a limitation of the study; however, in the context of the global COVID-19 pandemic, there is now ample evidence that sound qualitative research can be conducted at a distance using online information technology. Finally, it is important to highlight that the educational level of the interviewees is above the national average. A prior study suggests that in Mexico, around 39% of the women working in the formal sector and close to 50% of the women in reproductive ages working in the formal sector hold a technical or bachelor’s degree [35]. Our estimates are higher and might be explained by factors linked to the study design, such as the above-mentioned exclusion of rural and semi-urban employment sites and firms with less than 50 employees. In addition, our sample included sectors (i.e., university, government) that tend to have more employees with higher educational levels. The study design also overrepresented employees working in managerial and human resources areas. Hence, the generalization of the findings is limited by these factors.

In addition to the use of a unique framework, which was previously mentioned, one of the main strengths of this study is the inclusion of different actors in the workplace, which allows us to have a perspective both from the users themselves (current or potential), from those responsible for implementing them, such as managers and human resources staff, as well as from male employees. Similarly, we included a variety of types of worksites, by including public and private sector, as well as different lines of business.

In this study, several areas of opportunity were identified to promote a BF-friendly environment in the workplace which go beyond complying with current maternity protection legislation. Some recommendations at the public policy and workplace level are to develop and implement policies to prevent discrimination against pregnant women and mothers, particularly during BF; to raise awareness of maternity and paternity rights and benefits in the workplace and the processes for exercising them; to raise awareness among managers, human resources staff and staff, especially men, on the importance of BF and the support that can be provided to co-workers to continue BF; and to develop and implement workplace interventions that, in addition to promoting the establishment of lactation rooms, include strategies to provide tools and information to pregnant and lactating women in order to support the initiation and continuation of BF when returning to work. Ensuring compliance with labor laws to protect the health of women and children is of great importance. Lastly, there are some aspects of current Mexican legislation on maternity protection that need to be amended. For example, the option of reducing the working day by one hour (arriving one hour later or leaving one hour earlier), and time for milk extraction (two 30 min “rest” periods) in an adequate lactation room should not be mutually exclusive. This will facilitate women to continue BF by allowing them to spend more time with their young children and enhance milk production.

## 5. Conclusions

Mexico has legislation to protect maternal and child health once women return to the workplace. However, there are many contextual factors and underlying mechanisms in their implementation that can be improved in order to build a BF-friendly environment. One of the main underlying mechanisms and contextual factors is to raise awareness among directors, supervisors and human resources staff, co-workers and working mothers that the workplace is a space in which BF should be promoted, protected, and supported. To achieve this, complying with the provisions of the legislation is not sufficient, it is important to create a BF-friendly environment through various actions at the public policy level, as well as within workplaces.

## Figures and Tables

**Table 1:** General information of workplaces studied (February and November 2020) (*n* = 14).

Line of Business	Sector	Total Number of Employees ^1^	Number of Female Employees ^1^	Lactation Room Implemented
Mérida, Yucatán				
Shopping mall	Private	800	500	Yes
Public services	Public	65	40	No
Media and communication	Private	900	400	No
Health services	Private	425	260	No
Chihuahua, Chihuahua				
Media and communication	Private	230	150	No
Software development	Private	108	51	No
Educational services	Public	91	48	No
Purchase/sale of air conditioning and refrigeration	Private	137	37	No
Technology Development	Private	131	57	No
Manufacturing of industrial containers	Private	223	24	No
Guadalajara, Jalisco				
Educational services	Private	NA	NA	Yes
Tourism services	Private	146	51	Yes
Environment	Public	521	265	Yes
Monterrey, Nuevo León				
Beverage and tobacco industry	Private	471	38	Yes

NA: No information available. ^1^ Information reported by human resources personnel at the workplace.

**Table 2:** Study participant sociodemographic characteristics (*n* = 111).

Characteristic	Total
**Type of interviewee (%)**	
Beneficiaries or potential beneficiaries ^1^	49 (44.2)
Male employees	41 (36.9)
Managers and human resources personnel ^2^	21 (18.9)
**Sex (%)**	
Female	63 (56.8)
Male	48 (43.2)
**Marital Status (%)**	
Married/free union	93 (83.8)
Single	15 (13.5)
Divorced	3 (2.7)
**Interviewees’ age (Years) (SD)**	36.6 (±9.5)
**Education level (%)**	
Lower secondary ^3^ or Upper secondary incomplete ^4^	9 (8.1)
Upper secondary complete ^4^	11 (9.9)
Bachelor’s degree	67 (60.4)
Other ^5^	24 (21.6)

^1^ Female employees. ^2^ Includes Occupational Health staff, nurses, marketing managers and lactation promoters. ^3^ Grades 7 to 9 and Technician incomplete. ^4^ Grades 10 to 12. ^5^ Includes Master’s and Doctoral degrees.

**Table 3:** Representative quotes from semi-structured interviews based on the CMO Framework of Breastfeeding Interventions at the Workplace (*n* = 111).

Category	Subcategory	Original Quotations	English Translated Quotations ^1^
Context	Flexibility work schedule/workload	*“… Haz de cuenta, tienes ocho horas de trabajo y durante seis meses te dan media hora, ya sea que tú la tomes antes y después o que lo tomes durante el día. Entonces, yo lo que hacía era que… no sé si tú sepas, pero es doloroso tener la leche, entonces yo prefería tomar mis medias horas en los lactarios que salir o llegar más tarde porque era muy incómodo y doloroso para mí estar aguantando hasta salir. Entonces, tú elegías salir antes o tomar tus medias horas para tus extracciones…”* (Beneficiary or potential beneficiary, Guadalajara, [B01])	*“… You have eight hours of work and for six months they give you half an hour, whether you take it before and after or you take it during the day. So, what I did was… I don’t know if you know, but it is painful to have the milk, so I preferred to take my half hour in the lactation rooms than to leave or arrive later because it was very uncomfortable and painful for me to be waiting until I left. So, you could choose to leave earlier or take half an hour for your extraction…”* (Beneficiary or potential beneficiary, Guadalajara, [B01])
	Lactation services	*“… Pues, prácticamente se tiene la sala, se tienen las pláticas, lo coordinamos entrega de trípticos, lo hacemos sobre todo en el mes de agosto, que es la Semana de la Lactancia, son prácticamente como recordarle a la gente que existe (la sala). Obviamente también la damos a conocer en cada curso de inducción que se le da al personal nuevo. Entonces, son esas prácticas que se hacen, y se les da tanto a mujeres como a hombres, en nuestro curso de inducción y en el mes de la lactancia…”* (Manager and HR personnel, Guadalajara [RRHH])	*“… Well, we practically have the room, we have the talks, we coordinate the delivery of triptychs, we do it especially in the month of August, which is Breastfeeding Week, they are practically like a reminder to people that it exists (the lactation room). Obviously, we also make it known in each induction course given to new personnel. So, it’s those practices that are done, and it is given to both women and men, in our induction course and in the breastfeeding month…”* (Manager and HR personnel, Guadalajara [RRHH])
	Previous experience with BF	*[No support] “… Ahí sí porque no sabía, fíjate, porque yo le comenté a mi pediatra… bueno, ya cuando pasaron los años yo me di cuenta porque pensé que me había quedado sin leche, pero no, en realidad fue porque yo no me lo ponía constantemente y no producía, entonces yo desconocía eso, yo no sabía que tenía que estármelo poniendo a cada rato para que mi pecho produciera más leche, y ahora que lo supe dije “no, pues no es cierto, yo nunca me quedé sin leche, no hice que produjera leche…”* (Beneficiary or potential beneficiary, Chihuahua, [B04])	*[No support] “… I didn’t know, you see, because I told my pediatrician…well, when the years went by I realized it because I thought I had run out of milk, but no, in reality it was because I was not extracting constantly and I did not produce milk, so I did not know that, I did not know that I had to extract all the time so that my breast would produce more milk, and now that I knew I said “no, well it is not true, I never ran out of milk, I did not make it produce milk…”* (Beneficiary or potential beneficiary, Chihuahua, [B04])
	Distance between workplace and infant	*[Barrier] “…Pues vivo en una distancia lejana de aquí, entonces no había ni forma de ir a mi casa. Que viviera cerca sería lo ideal, agarro mi media hora, le doy leche, vengo y regreso, pero desgraciadamente así las cosas laborales…”* (Beneficiary or potential beneficiary, Mérida, [B02])	*[Barrier] “…Well, I live a long way from here, so there was no way to go home. If I lived close it would be ideal, I would take my half hour, give her breast milk, come, and go back, but unfortunately that’s the way things are at work…”* (Beneficiary or potential beneficiary, Mérida, [B02])
Mechanism	Awareness of the intervention	*[Promotion by workplaces] “… Pues mira, cuando abrimos la sala de lactancia, se abrieron más o menos al mismo tiempo en todas las plantas, yo estaba en Orizaba, me tocó que nos platicaron un poco, y cuando llegas a la planta te platican de las prestaciones, de las instalaciones. No es que tengas un entrenamiento mensual porque no se necesita, pero lo que sí he visto, es que cuando alguien se embaraza pues sí se le habla un poquito más a detalle…”* (Male employee, Monterrey, [H01])	*[Promotion by workplaces] “… Well, look, when we opened the lactation room, they opened more or less at the same time in all the stores, I was in Orizaba, they told us a little about it, and when you arrive at the store they tell you about the benefits and the facilities. It is not that you have monthly training because it is not necessary, but what I have seen is that when someone gets pregnant, they talk to them a little more in detail…”* (Male employee, Monterrey, [H01])
	Usage of the intervention	*“… Y ahorita mis extracciones las hago ahí… es la sala principal, tiene como una cocinita y en la cocinita tienen un cuartito donde guardan así como los insumos y ahí me encierro. Pido mi llave a la recepcionista, le digo “préstame la llave” y voy y me encierro y ya nadie puede entrar hasta que salgo…”* (Beneficiary or potential beneficiary, Chihuahua, [B03])	*“… And now I do my milk extractions there… it is the main room; it has a small kitchen and, in the kitchen, they have a little room where they keep the supplies and I lock up there. I ask the receptionist for my key, I say: “lend me the key” and I go and lock up and nobody can get in until I get out…”* (Beneficiary or potential beneficiary, Chihuahua, [B03])
Mechanism and Sub-Outcome	Workplace BF culture	*[Benefits beyond the law] “… Se tiene un grupo que lleva la jefa de Relaciones Laborales, que se llama “Women at XXX”, entonces hay reuniones de temas de liderazgo, pláticas… uno de los temas en algún momento fue el del lactario, el embarazo, igualdad de oportunidades, varios temas que tocaban, pláticas directas con el director, estaba muy padre. Entonces, nosotros tenemos muy claro el tema de fomentar la lactancia materna…”* (Manager and HR personnel, Monterrey, [RRHH01])	*[Benefits beyond the law] “… We have a group led by the head of Labor Relations, called “Women at XXX”, so there are meetings on leadership issues, talks… one of the topics at some point was the breastfeeding program, pregnancy, equal opportunities, several topics that were discussed, direct talks with the director, it was very cool. So, we are very clear on the issue of promoting breastfeeding…”* (Manager and HR personnel, Monterrey, [RRHH01])
	Physical environment	*[No physical space] “… Sí, porque todo es de vidrio, entonces era muy complicado porque era un local muy grande donde yo nada más estaba con dos hombres, éramos dos hombres y yo, entonces sí les decía: “muchachos, me voy a encerrar en el clóset, no pasen a la cocina, me voy a estar sacando leche, por si alguien pregunta por mí”, no hubo ningún problema. El clóset estaba muy chiquito […] tenía yo ahí mi silla, mi conector, me sacaba la leche, la guardaba en el refrigerador…”* (Beneficiary or potential beneficiary, Chihuahua, [B02])	*[No physical space] “… Yes, because everything is made of glass, so it was very complicated because where I was, it was a very big place, there were two men and I, so I told them: “guys, I’m going to lock myself in the closet, don’t go to the kitchen, I’m going to extract milk, in case someone asks for me”, there was no problem. The closet was very small […] I had my chair there, my connector, I extracted my milk, I kept it in the fridge…”* (Beneficiary or potential beneficiary, Chihuahua, [B02])
	Supervisor support	*[Inadequate support] “…creo que existe todavía un poco de machismo de los líderes, a lo mejor. Creo que es parte de no reconocer esto que es importante para el bienestar de sus empleados. Creo que va por ahí…”* (Male employee, Guadalajara, [H02])	*[Inadequate support] “…I think there is still a little bit of male chauvinism from the leaders, maybe. I think it’s part of not recognizing that this is important for the well-being of their employees. I think it goes that way…”* (Male employee, Guadalajara, [H02])
	Co-worker support	*[Perceived support] “… Igual la verdad fueron muy respetuosas, ayuda que sean puras mujeres creo que si hubiera algún hombre hubiera sido diferente […] había una mujer que también daba pecho y pues como que nos apoyamos un poco…”* (Beneficiary or potential beneficiary, Mérida, [B03])	*[Perceived support] “… The truth is that they were very respectful, it helps that they are all women, I think that if there had been a man it would have been different […] there was a woman who was also breastfeeding and we kind of supported each other a little bit…”* (Beneficiary or potential beneficiary, Mérida, [B03])

Note: For more details consult Supplementary Material S5. ^1^ Quotations have been translated from Spanish to English as expressed by the participants. CMO: Context-Mechanism-Outcome; BF: breastfeeding; HR: human resources.

## Data Availability

The data presented in this study are available on request from the corresponding author.

## Supplementary Materials

The following supporting information can be downloaded at: https://www.mdpi.com/article/10.3390/ijerph19042315/s1: Supplementary Material S1: Informed consent to participate in a research project; Supplementary Material S2: Semi-structured interview guide; Supplementary Material S3: Codebook-Implementation of breastfeeding policies at workplace in Mexico: Analysis of context using a realist approach; Supplementary Material S4: Study participant sociodemographic characteristics, by city, and Supplementary Material S5: Representative quotes from semi-structured interviews based on the CMO Framework of Breastfeeding Interventions at the Workplace.

## References

[1] Walters D.D., Phan L.T.H., Mathisen R. (2019). The cost of not breastfeeding: Global results from a new tool. Health Policy Plan..

[2] Smith P.H. (2018). Social Justice at the Core of Breastfeeding Protection, Promotion and Support: A Conceptualization. J. Hum. Lact..

[3] UNICEF (2019). State of the World’s Children 2019: Children, Food and Nutrition.

[4] World Health Organization, UNICEF (2009). Acceptable Medical Reasons for Use of Breast-Milk Substitutes.

[5] Rollins N.C., Bhandari N., Hajeebhoy N., Horton S., Lutter C.K., Martines J.C., Piwoz E.G., Richter L.M., Victora C.G. (2016). Why invest, and what it will take to improve breastfeeding practices?. Lancet.

[6] Neves P.A.R., Vaz J.S., Maia F.S., Baker P., Gatica-Domínguez G., Piwoz E., Rollins N., Victora C.G. (2021). Rates and time trends in the consumption of breastmilk, formula, and animal milk by children younger than 2 years from 2000 to 2019: Analysis of 113 countries. Lancet Child Adolesc. Health.

[7] González-Castell L.D., Unar-Munguía M., Quezada-Sánchez A.D., Bonvecchio-Arenas A., Rivera-Dommarco J. (2020). Situación de las prácticas de lactancia materna y alimentación complementaria en México: Resultados de la Ensanut 2018–2019. Salud Publica Mex..

[8] González de Cosío T., Escobar-Zaragoza L., González-Castell L.D., Rivera-Dommarco J.Á. (2013). Prácticas de alimentación infantil y deterioro de la lactancia materna en México TT—Infant feeding practices and deterioration of breastfeeding in Mexico. Salud Publica Mex..

[9] Vilar-Compte M., Hernández-Cordero S., Ancira-Moreno M., Burrola-Méndez S., Ferre-Eguiluz I., Omaña I., Pérez Navarro C. (2021). Breastfeeding at the workplace: A systematic review of interventions to improve workplace environments to facilitate breastfeeding among working women. Int. J. Equity Health.

[10] Unar-Munguía M., Lozada-Tequeanes A.L., González-Castell D., Cervantes-Armenta M.A., Bonvecchio A. (2021). Breastfeeding practices in Mexico: Results from the National Demographic Dynamic Survey 2006–2018. Matern. Child Nutr..

[11] U.S (2018). Department of Health and Human Services. The Business Case for Breastfeeding. https://www.womenshealth.gov/breastfeeding/breastfeeding-home-work-and-public/breastfeeding-and-going-back-work/business-case.

[12] Heymann J., Sprague A.R., Nandi A., Earle A., Batra P., Schickedanz A., Chung P.J., Raub A. (2017). Paid parental leave and family wellbeing in the sustainable development era. Public Health Rev..

[13] (2020). UNICEF Maternity and Paternity in the Workplace in Latin America and the Caribbean: A Review of National Policies for Paternity and Maternity Leave and Support to Breastfeeding in the Workplace Executive Summary. www.ipcig.org.

[14] (2014). Secretaría de Gobernación: Artículo 123 Constitución Política de los Estados Unidos Mexicanos (Ministry of the Government: Article 123 of the Political Constitution of the United Mexican States). http://www.ordenjuridico.gob.mx/Constitucion/articulos/123.pdf.

[15] (2015). Cámara de Diputados del H. Congreso de la Unión Ley Federal del Trabajo (Chamber of Deputies of the H. Congress of the Union Federal Labor Law). https://www.gob.mx/cms/uploads/attachment/file/156203/1044_Ley_Federal_del_Trabajo.pdf.

[16] Litwan K., Tran V., Nyhan K., Pérez-Escamilla R. (2021). How do breastfeeding workplace interventions work?: A realist review. Int. J. Equity Health.

[17] Wong G., Westhorp G., Greenhalgh J., Manzano A., Jagosh J., Greenhalgh T. (2017). Quality and reporting standards, resources, training materials and information for realist evaluation: The RAMESES II project. Heal. Serv. Deliv. Res..

[18] Galletta A. (2013). Mastering the Semi-Structured Interview and beyond: From Research Design to Analysis and Publication.

[19] Polit D.S., Beck C.T. (2010). Essentials of Nursing Research: Appraising Evidence for Nursing Practice.

[20] U.S (2013). Department of Labor Fact Sheet # 73: Break Time for Nursing Mothers under the FLSA. http://www.dol.gov/whd/regs/compliance/whdfs73.pdf.

[21] (1979). United States Department of Health, Education and Welfare. Protection of Human Subjects; Notice of Report for Public Comment. https://archive.epa.gov/osa/phre/web/pdf/belmont_report_frv44n76.pdf.

[22] Secretaría de Economía (2015). Norma Mexicana NMX-R-025-SCFI-2015 en Igualdad Laboral y No Discriminación. https://www.conapred.org.mx/userfiles/files/NMX-R-025-SCFI-2015_2015_DGN.pdf.

[23] Secretaría de Gobernación (2014). DECRETO por el que se adicionan y reforman diversas disposiciones de la Ley General de Salud; de la Ley Federal de los Trabajadores al Servicio del Estado, Reglamentaria del Apartado B) del artículo 123 Constitucional; de la Ley del Seguro Social; de la Ley del Instituto de Seguridad y Servicios Sociales de los Trabajadores del Estado; de la Ley para la Protección de los Derechos de Niñas, Niños y Adolescentes, y de la Ley General de Acceso de las Mujeres a una Vida Libre de Violencia (DECREE adding and amending various provisions of the General Health Law; the Federal Law of Workers in the Service of the State, Regulatory of Section B of Article 123 of the Constitution; the Social Security Law; the Law of the Institute of Security and Social Services of State Workers; the Law for the Protection of the Rights of Children and Adolescents, and the General Law on Women’s Access to a Life Free of Violence). https://www.dof.gob.mx/nota_detalle.php?codigo=5339161&fecha=02/04/2014.

[24] INEGI Resultados de la Encuesta Nacional de Ocupación y Empleo (2021). Nueva Edición (ENOEN)-Cifras Durante el Primer Trimestre de 2021 (INEGI Results of the National Occupation and Employment Survey. New Edition (ENOEN)-Figures during the First Quarter of 2021)..

[25] Victora C.G., Habicht J.P., Bryce J. (2004). Evidence-Based Public Health: Moving Beyond Randomized Trials. Am. J. Public Health.

[26] Pawson R., Greenhalgh T., Harvey G., Walshe K. (2005). Realist review—A new method of systematic review designed for complex policy interventions. J. Heal. Serv. Res. Policy.

[27] Chang Y.S., Harger L., Beake S., Bick D. (2021). Women’s and Employers’ Experiences and Views of Combining Breastfeeding with a Return to Paid Employment: A Systematic Review of Qualitative Studies. J. Midwifery Women’s Health.

[28] Scott V.C., Gigler M.E., Widenhouse J.M., Jillani Z.M., Taylor Y.J. (2020). A Socioecological Approach to Understanding Workplace Lactation Support in the Health Care Setting. Breastfeed. Med..

[29] Secretaría del Trabajo y Prevención Social (2016). Promueven Sectores Obrero y Empresarial Lactarios en Centros de Trabajo (Labor and Business Sectors Promote Lactation Rooms in Workplaces). Boletín No. 652. https://www.gob.mx/stps/prensa/promueven-sectores-obrero-y-empresarial-lactarios-en-centros-de-trabajo.

[30] Research Center for Equitable Development (2016). Índice País Amigo de la Lactancia Materna: Caso México 2020 (Becoming Breastfeeding Friendly Country Index: Mexico 2020 Case Study). https://equide.org/proyecto_bbf_2020_pb.pdf.

[31] Flacking R., Dykes F., Ewald U. (2010). The influence of fathers’ socioeconomic status and paternity leave on breastfeeding duration: A population-based cohort study. Scand. J. Public Health.

[32] Grandahl M., Stern J., Funkquist E.L. (2020). Longer shared parental leave is associated with longer duration of breastfeeding: A cross-sectional study among Swedish mothers and their partners. BMC Pediatr..

[33] Secretaría de Gobernación (2021). ACUERDO Mediante el cual se Aprueba la Modificación del Permiso de Paternidad, Previsto tanto en los Lineamientos en Materia de Recursos Humanos, Servicio Profesional y Personal de Libre Designación del INAI como en el Manual de Percepciones de los Servidores Públicos del Instituto Nacional de Transparencia, Acceso a la Información y Protección de Datos Personales (DECREE Approving the Modification of the Paternity Leave Provided for in the Guidelines on Human Resources, Professional Service and Free Appointment Personnel of INAI as well as in the Manual of Perceptions of Public Servants of the National Institute of Transparency, Access to Information and Protection of Personal Data). https://www.dof.gob.mx/nota_detalle.php?codigo=5631203&fecha=29/09/2021.

[34] Cools S., Fiva J.H., Kirkebøen L.J. (2015). Causal effects of paternity leave on children and parents Sara Cools BI Norwegian Business School BI Norwegian Business School. Scand. J. Econ..

[35] Vilar-compte M., Teruel G.M., Flores-peregrina D., Carroll G.J., Buccini S. (2020). Costs of maternity leave to support breastfeeding; Brazil, Ghana and Mexico. Bull. World Health Organ..

