# Supplementary material for: Implementation of Breastfeeding Policies at Workplace in Mexico: Analysis of Context Using a Realist Approach
Source: Int J Environ Res Public Health. 2022 Feb 17;19(4):2315. doi: 10.3390/ijerph19042315 (PMC8872172; doi:10.3390/ijerph19042315)
Supplement: Supplementary file 1 [file ijerph-19-02315-s001.zip › Supplementary Material S1.pdf]

UNIVERSIDAD IBEROAMERICANA
INFORMED CONSENT TO PARTICIPATE IN A RESEARCH PROJECT
BENEFICIARIES OR POTENTIAL BENEFICIARIES OF BREASTFEEDING POLICIES AT WORKPLACE

Diagnosis on the progress of policies in favor of breastfeeding in the workplace in Mexico

Dr. Sonia L. Hernández Cordero
Dr. Mireya Vilar Compte
Dr. Mónica M. Ancira Moreno
Izchel A. Cosío Barroso

Objective:
We are inviting you to participate in a research project designed to generate scientific evidence on the implementation of the "Guide for the Promotion of a Breastfeeding Culture in the Workplace" (Guide) and the development of policies that favor breastfeeding in the work environment, as well as to identify areas of improvement to positively impact policies in favor of breastfeeding in the workplace in Mexico, to generate recommendations that will feed back into the implementation and follow-up of the Guide.

Procedure:
Your participation implies answering a sociodemographic form in order to characterize the participants of this study, and to participate in an interview to learn about your experience regarding breastfeeding practices in your workplace. It will also be useful to know the suggestions or recommendations that you consider relevant to this issue.

Both the sociodemographic form and the interview will be applied and conducted by a trained and experienced research assistant. We would like to record the interview in order not to lose any detail. The recordings will then be transcribed word for word so that a complete and detailed analysis can be made.

Your participation will have a maximum duration of approximately 1 hour and will not interfere with your work schedule, in other words, it should not represent extra time for you. The interview can only be conducted at your place of work, in a private space where you feel comfortable and where there is no risk to confidentiality. It is important to note that you may stop the interview at any time.

Risks and benefits:
Your participation in this project does not involve any risk to you personally. Although there is always a risk of loss of confidentiality (for example, a person external to the study listening to the interview, mistakenly including the participant's name in the transcripts, or hacking), measures will be taken to reduce this risk, which is detailed below.

Although your participation in this project does not represent any benefit to you personally, it is expected that the results obtained will generate new knowledge for the implementation of policies that favor breastfeeding in the work environment.

Confidentiality:
Only members of the research team involved in this project will have access to your identification data and the information you provide to us. The Sociodemographic Record and the recordings will be identified with a folio number in such a way as to disassociate your name from the information you provide. In the transcription of your interview, names and any identifying information will be omitted; this information will be replaced by a general reference (Person A, Person B, etc.). All information collected will be stored in a computer with a security code, to which only the research team will have access. Consent will be stored separately.

The data collected during the study will be kept for no longer than 6 years. Recordings and identifying information will be destroyed once the research project ends.

When the results of the project will be published, your identification data will not be provided. Data obtained during the project may be shared with other researchers outside the project for the purpose of judging the conclusions obtained. This will be done as long as we ensure that your data privacy and confidentiality are protected.

Voluntary participation:
Your participation in the study is voluntary and you are entirely free to refuse to answer any of the questions or to withdraw your consent at any time, without any repercussions to you.

Questions:
If you have any questions or comments regarding the study, you may contact the responsible researcher: Dr. Sonia Hernández-Cordero, researcher at the Department of Health, *Universidad Iberoamericana*, Mexico City, at 55 5950 4000 ext. 4649, Monday through Friday from 9:00 to 15:00 hr or if you prefer you may write to her at *sonia.hernandez@ibero.mx.*

Signature:
I have read or have had read to me the information contained above. I have had the opportunity to resolve all my questions and I agree to voluntarily participate in this study.

______________________________	______________________________
Participant name	Participant signature
______________________________	______________________________
Witness name 1	Witness signature 1
______________________________	______________________________
Witness name 2	Witness signature 2

Date: ______________________________

UNIVERSIDAD IBEROAMERICANA
INFORMED CONSENT TO PARTICIPATE IN A RESEARCH PROJECT
MALE EMPLOYEES OF THE WORKPLACE

Diagnosis on the progress of policies in favor of breastfeeding in the workplace in Mexico

Dr. Sonia L. Hernández Cordero
Dr. Mireya Vilar Compte
Dr. Mónica M. Ancira Moreno
Izchel A. Cosío Barroso

Objective:
We are inviting you to participate in a research project designed to generate scientific evidence on the implementation of the "Guide for the Promotion of a Breastfeeding Culture in the Workplace" (Guide) and the development of policies that favor breastfeeding in the work environment, as well as to identify areas of improvement to positively impact policies in favor of breastfeeding in the workplace in Mexico, to generate recommendations that will feed back into the implementation and follow-up of the Guide.

Procedure:
Your participation implies answering a sociodemographic form in order to characterize the participants of this study, and to participate in an interview to learn about your experience regarding breastfeeding practices in your workplace. It will also be useful to know the suggestions or recommendations that you consider relevant to this issue.

Both the sociodemographic form and the interview will be applied and conducted by a trained and experienced research assistant. We would like to record the interview in order not to lose any detail. The recordings will then be transcribed word for word so that a complete and detailed analysis can be made.

Your participation will have a maximum duration of approximately 1 hour and will not interfere with your work schedule, in other words, it should not represent extra time for you. The interview can only be conducted at your place of work, in a private space where you feel comfortable and where there is no risk to confidentiality. It is important to note that you may stop the interview at any time.

Risks and benefits:
Your participation in this project does not involve any risk to you personally. Although there is always a risk of loss of confidentiality (for example, a person external to the study listening to the interview, mistakenly including the participant's name in the transcripts, or hacking), measures will be taken to reduce this risk, which is detailed below.

Although your participation in this project does not represent any benefit to you personally, it is expected that the results obtained will generate new knowledge for the implementation of policies that favor breastfeeding in the work environment.

Confidentiality:
Only members of the research team involved in this project will have access to your identification data and the information you provide to us. The Sociodemographic Record and the recordings will be identified with a folio number in such a way as to disassociate your name from the information you provide. In the transcription of your interview, names and any identifying information will be omitted; this information will be replaced by a general reference (Person A, Person B, etc.). All information collected will be stored in a computer with a security code, to which only the research team will have access. Consent will be stored separately.

The data collected during the study will be kept for no longer than 6 years. Recordings and identifying information will be destroyed once the research project ends.

When the results of the project will be published, your identification data will not be provided. Data obtained during the project may be shared with other researchers outside the project for the purpose of judging the conclusions obtained. This will be done as long as we ensure that your data privacy and confidentiality are protected.

Voluntary participation:
Your participation in the study is voluntary and you are entirely free to refuse to answer any of the questions or to withdraw your consent at any time, without any repercussions to you.

Questions:
If you have any questions or comments regarding the study, you may contact the responsible researcher: Dr. Sonia Hernández Cordero, researcher at the Department of Health, *Universidad Iberoamericana*, Mexico City, at 55 5950 4000 ext. 4649, Monday through Friday from 9:00 to 15:00 hr or if you prefer you may write to her at *sonia.hernandez@ibero.mx.*

Signature:
I have read or have had read to me the information contained above. I have had the opportunity to resolve all my questions and I agree to voluntarily participate in this study.

______________________________	______________________________
Participant name	Participant signature
______________________________	______________________________
Witness name 1	Witness signature 1
______________________________	______________________________
Witness name 2	Witness signature 2

Date: ______________________________

UNIVERSIDAD IBEROAMERICANA
INFORMED CONSENT TO PARTICIPATE IN A RESEARCH PROJECT
MANAGERS AND HUMAN RESOURCES PERSONNEL AT WORKPLACE

Diagnosis on the progress of policies in favor of breastfeeding in the workplace in Mexico

Dr. Sonia L. Hernández Cordero
Dr. Mireya Vilar Compte
Dr. Mónica M. Ancira Moreno
Izchel A. Cosío Barroso

Objective:
We are inviting you to participate in a research project designed to generate scientific evidence on the implementation of the “Guide for the Promotion of a Breastfeeding Culture in the Workplace” (Guide) and the development of policies that favor breastfeeding in the work environment, as well as to identify areas of improvement to positively impact policies in favor of breastfeeding in the workplace in Mexico, to generate recommendations that will feed back into the implementation and follow-up of the Guide.

Procedure:
Your participation implies answering a sociodemographic form in order to characterize the participants of this study, and to participate in an interview to learn about your experience regarding breastfeeding practices in your workplace. It will also be useful to know the suggestions or recommendations that you consider relevant to this issue.

Both the sociodemographic form and the interview will be applied and conducted by a trained and experienced research assistant. We would like to record the interview in order not to lose any detail. The recordings will then be transcribed word for word so that a complete and detailed analysis can be made.

Your participation will have a maximum duration of approximately 1 hour and will not interfere with your work schedule, in other words, it should not represent extra time for you. The interview can only be conducted at your place of work, in a private space where you feel comfortable and where there is no risk to confidentiality. It is important to note that you may stop the interview at any time.

Risks and benefits:
Your participation in this project does not involve any risk to you personally. Although there is always a risk of loss of confidentiality (for example, a person external to the study listening to the interview, mistakenly including the participant's name in the transcripts, or hacking), measures will be taken to reduce this risk, which is detailed below.

Although your participation in this project does not represent any benefit to you personally, it is expected that the results obtained will generate new knowledge for the implementation of policies that favor breastfeeding in the work environment.

Confidentiality:
Only members of the research team involved in this project will have access to your identification data and the information you provide to us. The Sociodemographic Record and the recordings will be identified with a folio number in such a way as to disassociate your name from the information you provide. In the transcription of your interview, names and any identifying information will be omitted; this information will be replaced by a general reference (Person A, Person B, etc.). All information collected will be stored in a computer with a security code, to which only the research team will have access. Consent will be stored separately.

The data collected during the study will be kept for no longer than 6 years. Recordings and identifying information will be destroyed once the research project ends.

When the results of the project will be published, your identification data will not be provided. Data obtained during the project may be shared with other researchers outside the project for the purpose of judging the conclusions obtained. This will be done as long as we ensure that your data privacy and confidentiality are protected.

Commitment:
The responsible investigator commits to send via email a generic report of the overall results of this study, between April and June 2020. This document will include all the workplaces and will have the sole purpose of informing about the findings and recommendations of the study, but does not imply any commitment of response, follow-up or presentation by the responsible researcher and/or the research team.

Email for submission: ____

Voluntary participation:
Your participation in the study is voluntary and you are entirely free to refuse to answer any of the questions or to withdraw your consent at any time, without any repercussions to you.

Questions:
If you have any questions or comments regarding the study, you may contact the responsible researcher: Dr. Sonia Hernández Cordero, researcher at the Department of Health, *Universidad Iberoamericana*, Mexico City, at 55 5950 4000 ext. 4649, Monday through Friday from 9:00 to 15:00 hr or if you prefer you may write to her at *sonia.hernandez@ibero.mx.*

Signature:
I have read or have had read to me the information contained above. I have had the opportunity to resolve all my questions and I agree to voluntarily participate in this study.

________________________________	________________________________
Participant name	Participant signature
________________________________	________________________________
Witness name 1	Witness signature 1
________________________________	________________________________
Witness name 2	Witness signature 2

Date: ________________________________
