# Supplementary material for: Implementation of Breastfeeding Policies at Workplace in Mexico: Analysis of Context Using a Realist Approach
Source: Int J Environ Res Public Health. 2022 Feb 17;19(4):2315. doi: 10.3390/ijerph19042315 (PMC8872172; doi:10.3390/ijerph19042315)
Supplement: Supplementary file 1 [file ijerph-19-02315-s001.zip › Supplementary Material S2.pdf]

UNIVERSIDAD IBEROAMERICANA

SEMI-STRUCTURED INTERVIEW GUIDE

BENEFICIARIES OR POTENTIAL BENEFICIARIES OF BREASTFEEDING POLICIES AT WORKPLACE

Objective of the instrument: to determine the level of satisfaction and knowledge about the existence of policies in favor of breastfeeding (BF) in the workplace by women in the reproductive age of the workplace.

GENERAL QUESTIONS:

1. How long have you been working here?
2. Could you tell us about the activities you do at work?
3. What are your regular working hours? (ask if there is overtime and how often)
4. What provisions do you have access to in this workplace?
   a. Are there any legal provisions that you do not have access to?
5. When you became pregnant, how much willingness did you find from the workplace to provide your maternity leave and other provisions such as time off for prenatal appointments?

A. BREASTFEEDING

1. What was the option you chose to take maternity leave: three weeks before and three (four) weeks after delivery / transfer of weeks postpartum?
2. What was the reason(s) for this?
3. Are you a new mother or do you have other children? (ask about the ages)
4. Did you breastfeed your child(ren)? (ask which ones yes and which ones no)
   a. If YES: How long did you breastfeed? (ask about motivations, barriers, etc.)
5. What are the reasons you decided to breastfeed? (or why you DID NOT breastfeed)
6. Do you know the benefits of BF for the baby and for the mothers?

a. How did you find out about these benefits?

7. How have you found the experience of breastfeeding at home? (ask about difficulties and ease)
8. At any time or with any of your children, did you decide or have the need to feed them with formula?
   a. IF YOU GIVED IT: Did you combine formula with breastfeeding? Did you include other food or drink?
   b. What was your experience with this form of feeding?

B. THE GUIDE AND ITS IMPLEMENTATION

Questions for BENEFICIARIES (workplaces that implemented the Guide)

1. When you returned to your job after taking maternity leave, what changes did you make in your work and home routine?
   a. How do you feed your baby since you returned to work? (Detect use of formula or

complementary/mixed feeding or exclusive breastfeeding)

2. What facilities did you find in the workplace to continue BF? (ask about the lactation room and other actions taken by the workplace)

3. Could you describe in detail what the workplace lactation room is like (facilities, hygienic conditions, schedule, amenities, shortcomings, etc.)?

4. How would you rate the operation of the breastfeeding center?

5. If there were no lactation room in this workplace, how would you be able to continue breastfeeding (e.g., milk extraction in the bathroom)? (Ask if you did this with previous children and what differences you find between the two experiences).

6. In addition to the lactation room, does the workplace have other actions to support breastfeeding?

7. What do you think are the reasons why the workplace decided to install the lactation room and (if applicable) to implement the aforementioned actions?

8. Do you think that the implementation of these recommendations has changed the general perception about the importance of BF in the people working in the workplace?

    a. What positive or negative attitudes did you perceive from your co-workers for having this benefit?

    b. What is the general perception of your co-workers about the importance of supporting and protecting BF?

9. Do you have any recommendations to improve the lactation room or (if applicable) the rest of the workplace's actions to protect and support BF?

10. Did you know that having protection and support for BF is a woman's right?

Questions for NON-BENEFICIARIES (due to access barriers of a personal nature or workplace omission, or due to NON-implementation of the Guide)

1. When you returned to your job, after taking maternity leave, what changes did you make in your work and home routine?
2. What facilities did you find in the workplace to continue BF?
3. And what have been the barriers?
4. What strategies have you used to continue BF (e.g., milk extraction in the bathroom)? Ask if you have done this with previous children and what differences you find between the two experiences).

a. IF YOU HAVE NOT SUCCESSFULLY CONTINUED BF: How do you feed your baby since returning to work? (Detect use of formula or complementary feeding)

5. What do you think are the reasons why the workplace does not have actions to support mothers during BF, for example, a lactation room?
6. What do you think is the general perception of those working in the workplace about the importance of supporting and protecting BF?
7. Did you know that having protection and support for BF is a woman's right?

UNIVERSIDAD IBEROAMERICANA

SEMI-STRUCTURED INTERVIEW GUIDE

MALE EMPLOYEES

Objective of the instrument: To identify the degree of knowledge of men about the existence of policies in favor of breastfeeding (BF) in the workplace.

GENERAL QUESTIONS:

1. How long have you been working here?
2. Could you tell us about the activities you do at work?
3. What are your regular working hours? (ask if there is overtime and how often)
4. What provisions do you have access to in this workplace?
    a. Did you have the opportunity to exercise your paternity leave?
    b. Are there any legal provisions that you do not have access to?

A. BREASTFEEDING

1. Do you have any daughters or sons? How many? (ask separately)
2. Did your partner breastfeed your child(ren)? (ask which ones yes and which ones no)
3. What are the reasons why your partner decided to breastfeed your child(ren)? (or why did he/she NOT breastfeed)
    a. Were you involved in this decision?
    b. Do you think this is a decision that only women can make? Why do you think so?
    c. In what ways do you think you supported or could have supported your partner to breastfeed your children? (IF YOU DO NOT HAVE CHILDREN raise it in the future)
4. Do you know the benefits of BF for the baby and for the mothers?
    a. How did you learn about these benefits?
5. Do you know what the workplace's policy on BF is?
6. What options do you know of for women to take maternity leave? (Ask about three weeks before and three (four) weeks after childbirth / transfer of weeks postpartum)

B. THE GUIDE AND ITS IMPLEMENTATION

Questions for MALE EMPLOYEES (workplaces that implemented the Guide)

1. Do you know what provisions are in place in the workplace for women to continue breastfeeding after their maternity leave? (ask about the lactation room and other actions carried out by the workplace)
2. Could you describe in detail what the workplace lactation room is like (facilities, hygienic conditions, schedule, amenities, shortcomings, etc.)?
3. How would you rate the operation of the breastfeeding center?
4. If there were no lactation room in this workplace, how do you think women would be able to continue breastfeeding? (e.g., milk extraction in the bathroom).
5. In addition to the lactation room, does the workplace have other actions to support breastfeeding?
6. What do you think are the reasons why the workplace decided to install the lactation room and (if applicable) to implement the aforementioned actions?
7. Do you think that the implementation of these recommendations has changed the general perception about the importance of BF in the people working in the workplace?
    a. What positive or negative attitudes did you perceive from your co-workers for having this benefit?

    b. What is the general perception of your co-workers about the importance of supporting and protecting BF?
8. Do you have any recommendations to improve the lactation room or (if applicable) the rest of the workplace's actions to protect and support BF?
9. In what way would you like to participate?

Questions for MEN (workplaces that did NOT implement the Guidelines)

1. Do you know what facilities are available in the workplace for women to continue breastfeeding after their maternity leave? (ask about the lactation room and other actions carried out by the workplace).
2. What barriers have you identified?
3. Do you know what strategies your coworkers develop to continue BF? (e.g., milk extraction in the bathroom)?
    a. IF YOU IDENTIFY THAT YOUR PARTNERS HAVE NOT SUCCESSFULLY CONTINUED BF: Do you know how your partners feed their babies when they return to work? (Detect use of formula or complementary feeding)
4. What do you think are the reasons why the workplace does not have actions to support mothers during BF, for example, a lactation room?
5. What do you think is the general perception of those working in the workplace about the importance of supporting and protecting BF?
6. How would you like to be involved in supporting and protecting BF?
7. Did you know that having protection and support for BF is a woman's right?

UNIVERSIDAD IBEROAMERICANA

SEMI-STRUCTURED INTERVIEW GUIDE

MANAGERS AND HUMAN RESOURCES PERSONNEL

Objective of the instrument: To identify the strengths and/or limitations among the workplaces that received the Guide that allowed or did not allow them to implement it.

A. BREASTFEEDING

GENERAL QUESTIONS

1. Could you tell us what is the average profile of the female employees currently working in the workplace? (ages, socioeconomic level, marital status, positions held).
2. Approximately how many of them have been mothers in the last year?
3. What does it mean for the workplace if one of its employees becomes pregnant? (ask about benefits, extraordinary hiring, absence from work, decreased performance, etc.)
4. What is the most common way in which employees decide to take their maternity leave: three weeks before and three (four) weeks after childbirth / transfer of weeks postpartum?
5. Do you know the reason why women decide to take more weeks after childbirth?

B. THE GUIDE AND ITS IMPLEMENTATION

Questions to ask the workplace that implemented the Guidelines

1. How did the workplace know about the existence of the Guide? (ask for a communication or visit from the Ministry of Labor and/or UNICEF, invitation, training, other means).
2. What was your and the workplace's initial reaction to this strategy? (ask for surprise, rejection, discomfort, relief, other)
3. What motivated the workplace to implement the recommendations of the Guide? (ask for benefits found for the workplace and for the beneficiaries).
4. At that time, what area/department/person was designated as responsible for implementation? At this time is there any change?
5. What challenges did you face in its implementation? (ask about economics, human knowledge, etc.) / How did you overcome them?
6. What factors do you think favored the implementation? (ask about management support, presence of women in high positions, support from the Ministry of Labor/UNICEF, size of the workplace, adaptation of any recommendation, others)?
7. Which of the Guide's recommendations do you consider have been better implemented and why?
8. Which of the Guide's recommendations have not been complied with and why? What is being done to achieve compliance?
9. In your opinion, how positive has the implementation of these recommendations been for both the workplace and its employees? (ask about job satisfaction, reputation of the workplace, long-term benefits of BF).
10. Do you think that the implementation of these actions has changed the general perception of the workplace about the importance of BF? How did it change?
11. How feasible is it for them to maintain these actions? (ask about facilitators and barriers)
12. In addition to the Guide, does the workplace implement other recommendations or strategies for the protection and support of the BF?
13. Do you have any recommendations for the successful implementation of the Guide by more workplaces?
14. Do you have any recommendations for improving the Guide?

Questions for workplaces that have NOT implemented the Guidelines

1. Can you tell us how you know about the Guide in your workplace? (communication or visit from the Ministry of Labor and/or UNICEF, invitation, training, etc.)
2. What was your and the workplace's initial reaction to this strategy? (probe for surprise, rejection, annoyance, relief, other)
3. What were the limitations or barriers to its implementation? (ask about economic aspects, human resources, knowledge, etc.)
4. What conditions or circumstances do you think would make it possible for this workplace to implement the Guide?
5. In your opinion, what benefits would the implementation of these recommendations bring to the workplace and its employees? (ask about job satisfaction, reputation of the workplace, long-term benefits of BF).
    a. If you do not see any benefits: why do you think there are no benefits?
6. Do you think that the implementation of these recommendations would change the general perception of the people working in the workplace about the importance of BF? In what sense or how?
7. Do you have any recommendations to ensure that workplaces such as yours could successfully implement the Guide? (ask if the cost of the lactation room is a relevant barrier)
