# Supplementary material for: Implementation of Breastfeeding Policies at Workplace in Mexico: Analysis of Context Using a Realist Approach
Source: Int J Environ Res Public Health. 2022 Feb 17;19(4):2315. doi: 10.3390/ijerph19042315 (PMC8872172; doi:10.3390/ijerph19042315)
Supplement: Supplementary file 1 [file ijerph-19-02315-s001.zip › Supplementary Material S4.pdf]

Study participant sociodemographic characteristics, by city

Characteristic	Chihuahua *n*=59	Guadalajara *n*=16	Mérida *n*=32	Monterrey *n*=4	Total *n*=111
**Type of interviewee (%)**					
Beneficiaries or potential beneficiaries [1]	30 (61.2)	5 (10.2)	14 (43.8)	0 (0.0)	49 (44.2)
Male employees	20 (48.8)	6 (14.6)	13 (40.6)	2 (50.0)	41 (36.9)
Managers and human resources personnel [2]	9 (42.9)	5 (23.8)	5 (15.6)	2 (50.0)	21 (18.9)
**Sex (%)**					
Female	34 (57.6)	8 (50.0)	20 (62.5)	1 (25.0)	63 (56.8)
Male	25 (42.4)	8 (50.0)	12 (37.5)	3 (75.0)	48 (43.2)
**Marital Status (%)**					
Married/free union	45 (76.3)	14 (87.5)	31 (96.9)	3 (75.0)	93 (83.8)
Single	12 (20.3)	2 (12.5)	0 (0.0)	1 (25.0)	15 (13.5)
Divorced	2 (3.4)	0 (0.0)	1 (3.1)	0 (0.0)	3 (2.7)
**Education level (%)**					
Lower secondary[3] or Upper secondary incomplete[4]	5 (8.5)	2 (12.5)	2 (6.2)	0 (0.0)	9 (8.1)
Upper secondary complete [4]	0 (0.0)	0 (0.0)	11 (34.4)	0 (0.0)	11 (9.9)
Bachelor’s degree	38 (64.4)	12 (75.0)	16 (50.0)	1 (25.0)	67 (60.4)
Other [5]	16 (27.1)	2 (12.5)	3 (9.4)	3 (75.0)	24 (21.6)

[1] Female employees
[2] Includes Occupational Health staff, nurses, marketing managers and lactation promoters.
[3] Grades 7 to 9 and Technician incomplete
[4] Grades 10 to 12
[5] Includes Master's and Doctoral degrees.
