# Supplementary material for: Implementation of Breastfeeding Policies at Workplace in Mexico: Analysis of Context Using a Realist Approach
Source: Int J Environ Res Public Health. 2022 Feb 17;19(4):2315. doi: 10.3390/ijerph19042315 (PMC8872172; doi:10.3390/ijerph19042315)
Supplement: Supplementary file 1 [file ijerph-19-02315-s001.zip › Supplementary Material S5.pdf]

Representative quotes from semi-structured interviews based on the CMO Framework of Breastfeeding Interventions at the Workplace ($n = 111$)

Category	Subcategory	Original quotations	English translated quotations [2]
Context	**Flexibility work schedule / workload**	"…Sí, me dieron una hora a escoger, ya ves que la Ley dice que es media hora para amamantar, me dieron la hora a escoger si entraba tarde o salía temprano, porque pues aquí no es como que puedas agarrar tu media hora y vayas a amamantar a tu niño o que haya un espacio para que te lo puedan traer. Sé que hay lugares que tienen lactario y puedes ir a extraerte leche en esas medias horas. Entonces, salía yo temprano, sin embargo, te puedo decir que había días que a mí no me dejaban salir a mi hora. El trabajo que tiene nuestra área a veces no lo permitía…" *(Beneficiary or potential beneficiary, Chihuahua, [B02])*	"... Yes, they gave me a time to choose, you know that the law says that it is a half hour to breastfeed, they gave me the time to choose whether to come in late or leave early, because here you can't take your half hour to go and breastfeed your child, nor is there a space for them to bring the baby to you. I know there are places that have a lactation room and you can go to express milk during those half hours. So, I would leave early, however, I can tell you that there were days that they would not let me leave at my time. The work that our area has sometimes did not allow it…" *(Beneficiary or potential beneficiary, Chihuahua, [B02])*
		"… La verdad no fue tan complicado, mi área es 100 por ciento operativa y vemos la planta y temas de proyectos, pero no tenemos operación a nuestro cargo. Además, la verdad es que la chava súper buena, entonces se organizó perfectamente bien, antes de irse de maternidad dejó perfectamente todo, yo cubrí algunas de las cosas que ella hacía y repartimos lo demás también entre los miembros del equipo y de otras áreas [...] después ella misma decidió que su mamá le podía ayudar a cuidar al bebé y ella quería como que regresar a la planta, entonces hice un horario con ella que a ella le convenía. Por ejemplo, me decía "quiero ir más días, pero prefiero ir de 10:00 de la mañana a 2:00 de la tarde porque es cuando me pueden cuidar al bebé y si no dormí bien me puedo quedar un poquito más" [...] y la verdad mientras estuviéramos de acuerdo los dos y que no hubiera ningún riesgo para ella, para el bebé, todo salió bien, la verdad no hubo ningún tema…" *(Male employee, Monterrey, [H01])*	"... The truth is that it wasn't so complicated, my area is 100 percent operational, we see the work floor and project issues, but we are not in charge of operations. Besides, the truth is that the girl was very good, so she organized herself perfectly well. Before leaving, for maternity leave, she left everything behind perfectly. I covered some of the things she used to do and we divided the rest among the members of the team and other areas [...] then she herself decided that her mother could help her take care of the baby and she wanted to return to the work floor, so I made a schedule with her that was convenient for her. For example, she told me: "I want to go more days, but I prefer to go from 10:00 in the morning to 2:00 in the afternoon because at that time they can take care of the baby and if I didn't sleep well I can stay a little longer" [...] And the truth is, as long as we both agreed and there was no risk for her or for the baby. Everything went well, there were no issues at all…" *(Male employee, Monterrey, [H01])*
		[No schedule flexibility] "… No, fue normal, yo no tuve eso de que podía salir antes o entrar más tarde. Era horario	[No schedule flexibility] "... No, it was normal, I didn't have that thing about leaving earlier or coming in later. It was a

	normal…” *(Beneficiary or potential beneficiary, Mérida, [B03])*	normal schedule…” *(Beneficiary or potential beneficiary, Merida, [B03])*
	“… Haz de cuenta, tienes ocho horas de trabajo y durante seis meses te dan media hora, ya sea que tú la tomes antes y después o que lo tomes durante el día. Entonces, yo lo que hacía era que… no sé si tú sepas, pero es doloroso tener la leche, entonces yo prefería tomar mis medias horas en los lactarios que salir o llegar más tarde porque era muy incómodo y doloroso para mí estar aguantando hasta salir. Entonces, tú elegías salir antes o tomar tus medias horas para tus extracciones…” *(Beneficiary or potential beneficiary, Guadalajara, [B01])*	“... You have eight hours of work and for six months they give you half an hour, whether you take it before and after or you take it during the day. So, what I did was... I don't know if you know, but it is painful to have the milk, so I preferred to take my half hour in the lactation rooms than to leave or arrive later because it was very uncomfortable and painful for me to be waiting until I left. So, you could choose to leave earlier or take half an hour for your extraction…” *(Beneficiary or potential beneficiary, Guadalajara, [B01])*
**Lactation services**	“…Mmm sí, te digo, el horario flexible, y también vienen muchas personas a hablarnos de todo lo relacionado con salud, maternidad y bebé…” *(Male employee, Chihuahua, [H02])*	“...Mmm yes, I tell you, the flexible schedule, and also many people come to talk to us about everything related to health, maternity and baby…” *(Male employee, Chihuahua, [H02])*
	“…Fue precisamente a partir de la necesidad, la demanda que había de las madres trabajadoras, pues aquí estábamos buscando un espacio que fuera privado, digno, limpio y sobre todo adecuado a las necesidades, ¿no? Entonces, tenemos un área de enfermería y a un costado de ahí está un espacio que se habilitó para la lactancia. Aquí por las instalaciones [...] hay algunas madres trabajadoras que cuentan con oficina privada, ellas antes de que estuviera el lactario ahí lo hacían, pero había muchas mamás que no, entonces a raíz de esa necesidad se creó. Ese espacio se encuentra desde hace aproximadamente unos cinco años…” *(Manager and HR personnel, Guadalajara, [RRHH02])*	“... It was precisely because of the need, the demand of the working mothers, that we were looking for a private, decent, clean and, above all, adequate space to meet their needs, right? So, we have a nursing area and next to it there is a space that was set up for breastfeeding. Here because of the installations [...] there are some working mothers who have a private office, they used to breastfeed before the lactation room was there, but there were many mothers who did not, so this was created as a result of that need. That space has been there for approximately five years…” *(Manager and HR personnel, Guadalajara, [RRHH02])*
	“…Tenemos el espacio, un seguimiento de acompañamiento cuando… en la clínica de aquí de cervecería ellas tienen su capacitación de cómo amamantar y las condiciones, cómo es un pezón, cómo pegarse al bebito y tenemos libros que están así en un librero muy bonito que tienen en la entrada como para estarse documentando, y yo tengo un seguimiento con	“...We have a space and a follow-up of accompaniment, which is a training on how to breastfeed and the conditions, what a nipple looks like, how to attach to the baby, and we have books that are in a very nice bookcase at the entrance so that they can be documented, and I have a follow-up with them, so we answer

	ellas, entonces, es el tema de responder a todas las dudas…” *(Manager and HR personnel, Monterrey, [RRHH01])*	all the doubts…” *(Manager and HR personnel, Monterrey, [RRHH01])*
	“… Pues, prácticamente se tiene la sala, se tienen las pláticas, lo coordinamos entrega de trípticos, lo hacemos sobre todo en el mes de agosto, que es la Semana de la Lactancia, son prácticamente como recordarle a la gente que existe (la sala). Obviamente también la damos a conocer en cada curso de inducción que se le da al personal nuevo. Entonces, son esas prácticas que se hacen, y se les da tanto a mujeres como a hombres, en nuestro curso de inducción y en el mes de la lactancia…” (*Manager and HR personnel, Guadalajara [RRHH])*	“... Well, we practically have the room, we have the talks, we coordinate the delivery of triptychs, we do it especially in the month of August, which is Breastfeeding Week, they are practically like a reminder to people that it exists (the lactation room). Obviously we also make it known in each induction course given to new personnel. So, it's those practices that are done, and it is given to both women and men, in our induction course and in the breastfeeding month…” *(Manager and HR personnel, Guadalajara [RRHH])*
	“… Una vez que salen de incapacidad estamos atentos a cuándo regresan porque ahí es cuando las captamos y hablamos con ellas la parte de lactancia materna, se les da una plática de lactancia materna, beneficios para ella, para el bebé, etc., y se le da un seguimiento, se le explica cuál es el periodo de lactancia y después de ese periodo, si ella necesita más días, cómo es que debe de pedirlo, porque puede solicitar más días del periodo que se da normalmente en la empresa. ¿Qué más? Se le ve mes con mes si no hay ningún problema durante la lactancia, si es así, se canaliza…” (*Manager and HR personnel, Mérida, [RRHH])*	“... Once they come off maternity leave, we are aware of when they return because at that time we contact them and talk to them about breastfeeding, we give them a talk on breastfeeding, benefits for them, for the baby, etc., and we provide follow-up, we explain to them what the breastfeeding period is and after that period, if they need more days, how should they request it, because they can request more days than the period that is normally given in the workplace. What else? She is monitored every month to see if there is no problem during lactation, if so, she is referred…” *(Manager and HR personnel, Mérida, [RRHH])*
**Previous experience with BF**	[No support] “…Sí, se quedó con hambre, bueno, se veía. Inclusive cuando yo me sacaba leche era muy poco lo que juntaba. A parte con la cesárea era muy cansado en una sola posición estar dando pecho y yo ya no aguantaba el dolor de la espalda y ella no quería despegarse del pecho, entonces opté por ayudarme con fórmula desde un principio…” (*Beneficiary or potential beneficiary, Chihuahua, [B01])*	[No support] “...Yes, he was hungry, well, you could see it. Even when I extracted my milk, I had very little. Besides, with the cesarean section it was very tiring to breastfeed in one position and I could not tolerate the pain in my back and she did not want to get off the breast, so I decided to help myself with formula from the beginning…” *(Beneficiary or potential beneficiary, Chihuahua, [B01])*
	[No support] “…Sí, de hecho, al principio sí fue pura leche materna y ya el tiempo que me iba preparando para regresar a trabajar, pues sí tuve que combinar la fórmula con lactancia,	[No support] “...Yes, in fact, at the beginning it was only breast milk and then when I was getting ready to go back to work, I had to combine formula with breastfeeding, that's how it was

así fue para los dos niños…" (*Beneficiary or potential beneficiary, Chihuahua, [B03])*

for both children…" *(Beneficiary or potential beneficiary, Chihuahua, [B03])*

[No support] "… Sí, a ella le tocó un poquito más de tiempo, con ella no tuve tanta leche como con el primer hijo, pero también me tocó afortunadamente darle, no lo que me hubiera gustado de seis meses o cuatro meses, por lo menos. Mucho tuvo que ver el tema del trabajo porque entre menos te estimules para sacarte leche, pues menos produces leche y entonces, ya cuando regresas a trabajar es bien complicado estar sacando leche. También ya no estaba tomando tantos líquidos y agua como cuando estuve incapacitada, que todo eso te ayuda a producir más leche. En el caso de mi trabajo, pues tocaba que era muy exigente el tipo de trabajo, pues no tenía mucho la posibilidad de escaparme a sacar leche o estar tome y tome agua, o sea, yo creo que sí influyó bastante el tema del trabajo…" (*Manager and HR personnel, Guadalajara, [RRHH02])*

[No support] "... Yes, she had a little more time, with her I did not have as much breast milk as with the first child, but fortunately I also had to give her, not as much as I would have, liked at least six months or four months. The work issue had a lot to do with it, because the less you stimulate yourself to express milk, the less milk you produce and then when you go back to work it is very complicated to express milk. I was also not drinking as many liquids and water as when I was on disability, which helps you produce more milk. In the case of my job, the type of work was very demanding, so I didn't have much chance to get away to express milk or to drink and drink water, I mean, I think the job had a lot of impact…" *(Manager and HR personnel, Guadalajara)*

[Success with BF] "… El primero pues la verdad, con el primero no trabajaba y pues estaba en la casa y sí le pude dar leche como casi dos años. Con este bebé pues tiene año dos meses exactamente, sigo dándole pecho cuando yo llego del trabajo y luego en la noche sigue tomando algo…" *(Beneficiary or potential beneficiary, Mérida, [B01])*

[Success with BF] "... The first one, the truth is, with the first one I didn't work and I was at home and I was able to give him milk for almost two years. With this baby, he is exactly one year and two months old, I continue to breastfeed him when I come home from work and then at night he continues to drink something..." *(Beneficiary or potential beneficiary, Mérida, [B01])*

[Success with BF] "…Mi mamá, con mi primer bebé. Es que con mi primera bebé aprendí muchas cosas, mi mamá me enseñó, me compró el extractor, o sea, realmente que el cuerpo de la mujer es maravilloso porque ni batallé para darles pecho, siempre me salió mucha leche, siempre, siempre, siempre, hasta la fecha. Entonces, yo misma fui aprendiendo y con, pues, la compañía de mi mamá. Y ahora que nació mi segundo bebé pues, yo me alivié el 29 de septiembre, para el 2 de octubre ya estaba haciendo mi banco de leche y, prácticamente, sobre la marcha aprende uno…" (*Beneficiary or potential beneficiary, Chihuahua, [B04])*

[Success with BF] "...My mom, with my first baby. With my first baby I learned many things, my mom showed me, she bought me the breast pump, I mean, a woman's body is really wonderful because I didn't even have to fight to breastfeed, I always had enough milk, always, always, always, to this day. So, I was learning by myself and with, well, my mother's workplace. And now that my second baby was born, well, I was relieved on September 29, by October 2 I was already doing my milk bank and, practically, you learn as you go along…" *(Beneficiary or potential beneficiary, Chihuahua, [B04])*

	[No support] "... Ahí sí porque no sabía, fíjate, porque yo le comenté a mi pediatra... bueno, ya cuando pasaron los años yo me di cuenta porque pensé que me había quedado sin leche, pero no, en realidad fue porque yo no me lo ponía constantemente y no producía, entonces yo desconocía eso, yo no sabía que tenía que estármelo poniendo a cada rato para que mi pecho produciera más leche, y ahora que lo supe dije "no, pues no es cierto, yo nunca me quedé sin leche, no hice que produjera leche…" (*Beneficiary or potential beneficiary, Chihuahua, [B04])*	[No support] "... I didn't know, you see, because I told my pediatrician....well, when the years went by I realized it because I thought I had run out of milk, but no, in reality it was because I was not extracting constantly and I did not produce milk, so I did not know that, I did not know that I had to extract all the time so that my breast would produce more milk, and now that I knew I said "no, well it is not true, I never ran out of milk, I did not make it produce milk…" *(Beneficiary or potential beneficiary, Chihuahua, [B04])*
**Distance between workplace and infant**	**[**Enabling factor] "… la hora de lactancia la tomaba media hora antes de entrar a trabajar y salía media hora antes de mi horario de trabajo. También tengo dos horas de comida, vivo muy cerca entonces iba a mi casa, eso igual me ayudaba…" *(Beneficiary or potential beneficiary, Mérida, [B01])*<br><br>[Enabling factor] "… Igual me traían a la bebé aquí al trabajo porque te digo que tengo la cercanía o sea eso me ayuda mucho, pero igual sí he utilizado en algunas ocasiones el lactario…" *(Beneficiary or potential beneficiary, Mérida, [B01])*<br><br>**[**Barrier] "…Pues vivo en una distancia lejana de aquí, entonces no había ni forma de ir a mi casa. Que viviera cerca sería lo ideal, agarro mi media hora, le doy leche, vengo y regreso, pero desgraciadamente así las cosas laborales…" *(Beneficiary or potential beneficiary, Mérida, [B02])*<br><br>[Barrier] "...pero ahora a una o dos horas de distancia, pues no va a darle al niño o la niña, no va a poder darse una escapada a su casa…" *(Manager and HR personnel, Guadalajara, [RRHH01])*	[Enabling factor] "... I took the breastfeeding hour half an hour before going to work and I left half an hour before my work schedule. I also have two hours for lunch, I live very close so I would go home, that also helped me..." *(Beneficiary or potential beneficiary, Mérida, [B01])*<br><br>[Enabling factor] "... They used to bring my baby here to work because I have the proximity, that is, that helps me a lot, but I have also used the lactation room on some occasions..." *(Beneficiary or potential beneficiary, Mérida, [B01])*<br><br>[Barrier] "...Well, I live a long way from here, so there was no way to go home. If I lived close it would be ideal, I would take my half hour, give her breast milk, come and go back, but unfortunately that's the way things are at work…" *(Beneficiary or potential beneficiary, Mérida, [B02])*<br><br>[Barrier] "... But now an hour or two away, because she's not going to breastfed the boy or the girl, she's not going to be able to go home [in such a short time]..." *(Manager and HR personnel, Guadalajara, [RRHH01])*

		"... Entonces, sí debería ser el derecho de pedir que haya un lactario para que tú puedas realizarlo, pero si nos vamos a esos puntos, nosotros como clínica qué pediríamos mejor entre un lactario o una estancia, pues creo que la estancia, sería hasta más fácil [...] tú estando aquí laborando "¿sabes qué?, siento que ya me llené" que la Clínica nos pueda proporcionar ¿qué será?, media hora o una hora, de poder ir, darle lechita al bebé y regresar [...] Te dan a tu bebé, estás en contacto con él, viendo que en realidad esté bien, le das sus dos tomas. Tú estás feliz, no estás incómoda, bebé lleno, y sigues cumpliendo con tus labores. Eso es lo que en realidad debería de ser, ¿no?, pero pues... se vale soñar…" *(Beneficiary or potential beneficiary, Mérida, [B04])*	"... So, yes, it should be the right to ask for a lactation room so that you can do it, but if we go to those points, we as a clinic would ask for a lactation room or a daycare, because I think the daycare would be easier [...] you being here working, "you know what? I feel that my breasts are full" that the clinic can provide us, what would it be?, half an hour or an hour to be able to go (to the daycare), give milk to the baby and come back [...] They give you your baby, you are in contact with him, seeing that he is well, you give him his two feedings. You are happy, you are not uncomfortable, the baby is full, and you go on with your work. That's what it should really be, right?, but well... it's fair to dream..." *(Beneficiary or potential beneficiary, Mérida, [B04])*
		"... se refiere a que la empresa tiene que ayudarte a ti como mujer a seguir con tu lactancia [...] quitarles una hora para que puedan salir, o ponerles su lactario y un refri para que puedan extraerse leche [...] porque en el baño como que estaría raro, ¿no? [...] como que hasta uno por higiene. Ahora hasta hay empresas que tienen como guardería abajo, tú puedes subir y bajar. Entonces pues sí, es más como que intentar invitar mucho a las empresas para que cuiden más, pues de sus mujeres, y de los padres también, porque hay padres que apoyan mucho la lactancia en el trabajo…" *(Beneficiary or potential beneficiary, Chihuahua, [B07])*	*"*... it means that the workplace has to help you as a woman to continue breastfeeding [...] give them an hour so that they can go out, or provide them a lactation room and a refrigerator so that they can extract their milk [...] because it would be strange in the bathroom, wouldn't it? [...] it would be weird, wouldn't it?, even for hygiene reasons. Now there are workplaces that even have a daycare downstairs, you can go up and down. So, yes, it is more like trying to invite workplaces to take more care of their women, and of the fathers too, because there are fathers who are very supportive of breastfeeding at work…" *(Beneficiary or potential beneficiary, Chihuahua, [B07])*
**Mechanism**	**Awareness of the intervention**	[Unawareness of workplace policies] "... (se le preguntó si había leído o le habían dicho que es un derecho de la mujer que en la empresa se proteja y apoye la lactancia materna) Eso sí no sabía [risas], no había escuchado nada…" *(Beneficiary or potential beneficiary, Chihuahua, [B01])*	[Unawareness of workplace policies] "... (was asked if she had read or been told that it is a woman's right to have breastfeeding protected and supported in the workplace) I didn't know that [laughs], I hadn't heard anything...." *(Beneficiary or potential beneficiary, Chihuahua, [B01])*
		"… Pues no veo… No he visto en mi departamento que les digan algo, al contrario les prestan un apoyo en ese sentido, de que, por ejemplo, ellas trabajan un periodo de ocho horas ¿no?, La jornada laboral y les dan la posibilidad de salir una hora antes para poder ejercerla…" *(Male employee, Mérida, [H01])*	"... Well, I don't see...I haven't seen in my department that they tell them anything, on the contrary, they give them support in that sense, that, for example, they work a period of eight hours, right, the working day, and they give them the possibility of

"… Pues las que te menciono. Les dan tiempo para salir del trabajo para ir a atender a sus bebés, a medio día y salir más temprano para que puedan acudir con sus hijos. Esas son las que yo sé, o sea, desconozco si hay alguna otra…" *(Male employee, Chihuahua, [H02])*

[Unawareness of workplace policies] "… Pues no así directamente por la empresa, como había dicho antes […] se presta que las mujeres se tomen un tiempo para que se extraigan la leche y así, pero desconozco la política de la empresa…" *(Male employee, Chihuahua, [H03])*

[Promotion by workplaces] "… No, de hecho se mantiene informado a todo el personal de que se hizo la reubicación de la sala, y no solo por el personal interno o por los clientes internos que son nuestros empleados, sino por si también en algún momento fuera necesario para los huéspedes, que ha pasado ya una vez hace un par de años, pues que estén enterados, si te preguntas tengas la información de poder decir: "sí, sí contamos en la empresa con sala de lactancia, sí puede utilizarla", por eso es que se le da un mantenimiento constante…" *(Beneficiary or potential beneficiary, Guadalajara, [B02])*

[Promotion by workplaces] "… Pues mira, cuando abrimos la sala de lactancia, se abrieron más o menos al mismo tiempo en todas las plantas, yo estaba en Orizaba, me tocó que nos platicaron un poco, y cuando llegas a la planta te platican de las prestaciones, de las instalaciones. No es que tengas un entrenamiento mensual porque no se necesita, pero lo que sí he visto, es que cuando alguien se embaraza pues sí se le habla un poquito más a detalle…" *(Male employee, Monterrey, [H01])*

[Promotion by workplaces] "… Sí, durante la inducción, que es muy general, se habla de todas las prestaciones, el tema de horarios, de reglamento interior y todo así a grandes rasgos

leaving an hour earlier to be able to exercise it…" *(Male employee, Merida, [H01])*

"... Well, the ones I mention. They give them time to leave work to go to take care of their babies, in the middle of the day and leave earlier so that they can go with their children. Those are the ones that I know of, I mean, I don't know if there are any others…" *(Male employee, Chihuahua, [H02])*

[Unawareness of workplace policies] "... Well, not directly by the workplace, as I said before [...] it is possible for women to take some time to extract their milk, but I don't know the workplace policy…" *(Male employee, Chihuahua, [H03])*

[Promotion by workplaces] "... No, in fact, all the staff is kept informed that the lactation room has been relocated, and not only for the internal staff or for the internal clients who are our employees, but also in case it is necessary for the guests, which has already happened once a couple of years ago. It is important that they are aware so that if someone asks them they have the information to be able to say: "yes, we have a lactation room in the workplace, you can use it", that's why it is constantly maintained…" *(Beneficiary or potential beneficiary, Guadalajara, [B02])*

[Promotion by workplaces] "... Well, look, when we opened the lactation room, they opened more or less at the same time in all the stores, I was in Orizaba, they told us a little about it, and when you arrive at the store they tell you about the benefits and the facilities. It is not that you have monthly training because it is not necessary, but what I have seen is that when someone gets pregnant, they talk to them a little more in detail…" *(Male employee, Monterrey, [H01])*

	datos generales que se necesitan saber como colaborador y en esa información tratan el tema de la sala de lactancia…” *(Male employee, Guadalajara, [H01])*	[Promotion by workplaces] “…Yes, during induction, which is very general, it talks about all the benefits, schedules, internal regulations and all the general information that you need to know as an employee and in this information they talk about the lactation room…” *(Male employee, Guadalajara, [H01]).*
	[Promotion by workplaces] “… En un primer momento lo dimos a conocer por correo electrónico, pusimos alguna foto. Se hizo responsable a una persona para que lleve el registro para que ahí se anoten, hay lugar para dos entonces para que sepan si hay cupo… el cupo no ha sido problema, me parece [...] ya posteriormente con la Norma en marcha ya teníamos que impulsarlo todavía más, con el conocimiento de la Norma de difundir no solo la existencia del lactario sino difundir o recomendar el uso de la lactancia o difundir las medidas que hay que tomar para la lactancia o difundir algunas cuestiones de tipo científico del beneficio de la lactancia. Cada cierto tiempo se difunde a todo el personal, hombres y mujeres del beneficio de la lactancia…” *(Manager and HR personnel, Guadalajara, [RRHH01])*	[Promotion by workplaces] “...At first we made it known by email, we put some pictures. A person was made responsible for keeping the register so that they could sign up, there is room for two so that they would know if there is space... space has not been a problem, I think [...] later on, with the Norm in place, we had to promote it even more, with the knowledge of the Norm to disseminate not only the existence of the lactation room but also to disseminate or recommend the use of breastfeeding or disseminate the measures to be taken for breastfeeding or disseminate some scientific questions about the benefits of breastfeeding. At a certain time, all personnel, both men and women, are informed of the benefits of breastfeeding…” *(Manager and HR personnel, Guadalajara, [RRHH01])*
**Usage of the intervention**	“… Yo pensaría que unas cinco mujeres han utilizado el lactario, más o menos. Este año fueron como unas cinco literal, pero por tema de Covid no se ha usado […] Yo tuve en mi equipo una nutrióloga y este año se alivió de su bebé y estuvo en cuarentena, no le tocó usar el lactario tampoco, a quien le pudo tocar fue a una empleada previa que tuve y ella sí lo usó […] Pero eso sí, si se les da oportunidad que puedan tener su horario de lactancia o puedan tener su horario flexible, buscan usar el lactario…” (*Manager and HR personnel, Monterrey, [RRHH01])*	“... I would think that about five women have used the lactation room more or less. This year there were literally about five, but because of Covid it has not been used [...] I had a nutritionist in my team and this year she got relief from her baby and was in quarantine, she wasn't able to use the lactation room either, the one who was able to use it was a previous employee I had and she did use it [...] But yes, if they are given the opportunity to have their breastfeeding times or have a flexible schedule, they try to use the lactation room…” *(Manager and HR personnel, Monterrey, [RRHH01])*
	“… Lo único que he escuchado de una compañera que tiene un niño recién nacido y sé que utiliza esa hora, esas horas de lactancia, porque nosotros comemos una hora, pero a ellas les dan dos horas, entonces tienen tiempo de estar allá con el	“... The only thing I have heard from a female co-worker who has a newborn child and I know that she uses that hour, those breastfeeding hours, because we eat one hour, but they get two hours, so they have time to be there with the newborn, but then

recién nacido, pero pues tienen que hacer el tramo, el viaje a su casa…” *(Male employee, Chihuahua, [H03])*

they have to make the journey, the travel home…” *(Male employee, Chihuahua, [H03])*

“… El lactario está ubicado a un lado de la enfermería, entonces la chica, la paramédico que está ahí, ella me apoya con abrirlo y cerrarlo, entonces desde las 9:00 de la mañana hasta las 6:00 de la tarde está siempre abierto. Por lo mismo que se tiene un espacio de dos cubículos están privados con una mampara. Entonces, cuando regresan me hacen una consulta de cómo lo pueden utilizar, les digo que es un espacio abierto y privado en el sentido de que nadie las va a molestar, que solamente se les pide se registren en la bitácora cada vez que lo vayan a utilizar. Realmente, como son alrededor de tres, cuatro mamás, casi siempre ya saben ellas los horarios que se van a manejar, entonces hasta el momento no he tenido conflicto con los horarios porque hay espacio para que dos mamás al mismo tiempo se puedan estar extrayendo la leche [...] No necesariamente llevamos un control muy estricto porque hasta el momento se han dado las facilidades para que las madres trabajadoras puedan acudir sin que se traslapen sus horarios…” *(Manager and HR personnel, Guadalajara, [RRHH02])*

“... The lactation room is located next to the infirmary, so the girl, the paramedic who is there, helps me to open and close it, so from 9:00 in the morning until 6:00 in the evening it is always open. For the same reason that there is a space of two cubicles, they have a private partition. So, when they come back they ask me how they can use it, I tell them that it is an open and private space in the sense that no one will bother them, that they only have to register in the logbook every time they are going to use it. Actually, since there are about three or four mothers, they almost always know the schedules they are going to use, so I didn't have any conflict with the schedules so far because there is room for two mothers to express milk at the same time [...] We don't necessarily keep a very strict control because so far we have provided the facilities so that working mothers can attend without overlapping their schedules…” *(Manager and HR personnel, Guadalajara, [RRHH02])*

“… Sí claro lo pueden utilizar sin problema, acuérdate que ellas tienen también sus medias horas […] pues finalmente es su decisión, una chica estuvo fuera porque fue considerada como vulnerable por estar lactando, entonces cuando regresó le quedaban dos meses y el acuerdo fue de que se fuera una hora antes, pero, si ella decide, puede ir a utilizar la sala de lactancia sin ningún problema, de hecho, no hay nadie que cuestiona ese tema, solamente hay un registro…” (*Manager and HR personnel, Monterrey, [RRHH02])*

“... Yes, of course they can use it without any problem, remember that they also have their half hours [...] in the end it is their decision, one girl was out because she was considered vulnerable because she was breastfeeding, so when she returned she had two months left and the agreement was that she should leave an hour earlier, but, if she decides, she can go and use the lactation room without any problem, in fact, there is no one who questions this issue, there is only a record…” *(Manager and HR personnel, Monterrey, [RRHH02])*

“… Y ahorita mis extracciones las hago ahí... es la sala principal, tiene como una cocinita y en la cocinita tienen un cuartito donde guardan así como los insumos y ahí me encierro. Pido mi llave a la recepcionista, le digo "préstame la llave" y voy y me encierro y ya nadie puede entrar hasta que

“... And now I do my milk extractions there... it is the main room, it has a small kitchen and in the kitchen they have a little room where they keep the supplies and I lock up there. I ask the receptionist for my key, I say: "lend me the key" and I go and

		salgo…” *(Beneficiary or potential beneficiary, Chihuahua, [B03])*<br><br>“... Nos ha tocado llegar a lactarios que anteriormente estaban inaugurados y ahora son bodegas; dos, la falta de uso, y esa falta de uso también es culpa de nosotras como mujeres que es lo que yo te comentaba, a veces las mismas mujeres pedimos cosas pero no las usamos, no las aplicamos y no las promovemos…” (*Manager and HR personnel, Mérida, [RRHH]*)	lock up and nobody can get in until I get out…” *(Beneficiary or potential beneficiary, Chihuahua, [B03])*<br><br>“... we have had to go to lactation rooms that were previously opened and are now storage rooms. Two, the lack of use, and that lack of use is also the fault of us as women, which is what I was telling you. Sometimes we women ourselves ask for things but we don't use them, we don't apply them and we don't promote them…” *(Manager and HR personnel, Mérida, Mérida, [RRHH])*
**Mechanism and Sub-Outcome**	**Workplace BF culture**	[Importance of a BF-friendly environment in the workplace] “… Sí, a lo mejor más promoción en eso, que no nada más uno, todos nos concienticemos, no nada más uno como mamá o las que van a ser mamás sino todos estemos conscientes de este tema para que haya más apertura y, sobre todo, que... otra vez repito, más conscientes al tema de que pues podemos amamantarlo aquí o que puedes salir, a lo mejor se te maneja que sí puedes salir, pero no tienes la confianza en hacerlo porque a lo mejor te vayan a decir "no", o a lo mejor para la otra no te contraten porque vas a ser mamá. Que puedas sentirte tú con la confianza o la seguridad de "ya está el espacio", se promueve y ya, con confianza lo hago y ejerzo el derecho…” *(Beneficiary or potential beneficiary, Chihuahua, [B01])*<br><br>[Incentives enabling BF-friendly environment in the workplace] “… No, no, para nada porque está debidamente estipulado. De hecho, nosotros estamos certificados por la Norma de igualdad en donde este certificado nos apoya, nos permite la difusión, a favorecer los espacios y, sobre todo, a sensibilizar a los jefes y a las jefas de que deben darles las facilidades a las madres trabajadoras, ya que este es un derecho hacia los menores, entonces lo que promovemos es ese derecho de la lactancia hacia los hijos…” (*Manager and HR personnel, Guadalajara, [RRHH02])*	[Importance of a BF-friendly environment in the workplace] “... Yes, maybe more promotion in that, that not only one, we all become aware, not only one as a mother or those who are going to be mothers, but that we all become aware of this issue so that there is more openness. Above all, we should be more aware that we can breastfeed here or that you can go out, maybe you can go out, but you do not have the confidence to do it because maybe they will say "no", or maybe they will not hire you for the next one because you are going to be a mother. That you can feel confident or sure that "the space is there", it is promoted and now, with confidence I do it and I exercise my right…” *(Beneficiary or potential beneficiary, Chihuahua, [B01])*<br><br>[Incentives enabling BF-friendly environment in the workplace] “... No, no, not at all, because it is properly stipulated. In fact, we are certified by the Equality Norm where this certificate supports us, allows us to disseminate, to promote spaces and, above all, to raise awareness among managers that they should provide facilities for working mothers, since this is a right for children, so what we promote is the right to breastfeed their children…” *(Manager and HR personnel, Guadalajara, [RRHH02])*

[Incentives enabling BF-friendly environment in the workplace] “... Nos hicieron saber que existía otro que se llamaba “Empresa Familiarmente Responsable”, y dentro de las directrices, porque ya hacíamos nosotros los cursos de la lactancia, dábamos la información y las capacitaciones, le explicábamos a la gente la importancia de esto, ¿no?, pero no teníamos un espacio como tal para brindar el apoyo a una persona que llegara a necesitarlo. Entonces, ahondando en este reconocimiento fue en donde nos dimos cuenta que uno de los puntos que nos pedían era tener una sala de lactancia y sí nos gustó mucho la idea porque ya teníamos pensado algo así, pero no terminaba de cuajar la idea, entonces la Secretaría nos dio como todas las directrices para poderlo poner de una manera ordenada y darle ese protocolo formal…” *(Male employee, Guadalajara, [H01])*	[Incentives enabling BF-friendly environment in the workplace] “... They let us know that there was another (recognition) called "Family Responsible Company", and within the guidelines, because we were already giving courses on breastfeeding, we were providing information and training, we were explaining to people the importance of this, but we did not have a space as such to provide support to a person who needed it. So, when we looked into this recognition, we realized that one of the things they asked us to do was to have a lactation room, and we really liked the idea because we had already thought of something like that, but the idea was not quite ready. The Ministry gave us all the guidelines to put it in an orderly manner and give it that formal protocol…” *(Male employee, Guadalajara, [H01])*
[(lack) Incentives enabling BF-friendly environment in the workplace] “... Hemos hablado con directivos, te dicen que está muy padre la iniciativa, pero no te vuelven a llamar; cuando tú los vuelves “a molestar”, por así decirlo, una de las cosas que te comentan es que “¡chispas!, es que tengo que pasar ese proyecto a que me lo aprueben porque cuesta, si fuera un proyecto que no costara no habría problema, yo doy el banderazo”, pero es una iniciativa que cuesta porque si no tengo el espacio lo tengo que construir y si lo tengo, lo tengo que habilitar”...” (*Manager and HR personnel, Mérida, [RRHH]*)	[(lack) Incentives enabling BF-friendly environment in the workplace] “... We have talked to managers, they tell you that the initiative is very cool, but they don't call you back; when you go back to "bother" them, so to speak, one of the things they tell you is that “ups!, I have to submit this project for approval because it costs, if it were a project that did not cost anything there would be no problem, I would give the green light, but it is an initiative that costs because if I do not have the space, I have to build it and if I have it, I have to enable it”...” (*Manager and HR personnel, Mérida, [RRHH]*)
[(lack) Incentives enabling BF-friendly environment in the workplace] “...nuestro principal deber es la academia, necesitamos cubículos para los profesores, necesitamos salones para nuestros estudiantes… se han generado una serie de prioridades, es por eso, me parece, que el lactario está tomando forma hasta ahora…” (*Manager and HR personnel, Guadalajara, [RRHH01])*	[(lack) Incentives enabling BF-friendly environment in the workplace] “...our main duty is the academy, we need office spaces for teachers, we need classrooms for our students... a series of priorities have been generated, that is why, it seems to me, that the lactation room is taking shape so far…” (*Manager and HR personnel, Guadalajara, [RRHH01])*
[Importance of a BF-friendly environment in the workplace] “… Pues yo creo que mucho, porque además de que las mamás	[Importance of a BF-friendly environment in the workplace] “... Well, I think it is very important, because in addition to the fact

no sentimos incluidas, en la cuestión de la compatibilidad de la vida laboral y familiar, yo creo que en el sentido de pertenencia al lugar de trabajo. Si ha favorecido al ambiente laboral y se sienten más acogidas las mamás. La verdad es que sí fue bien visto tanto por las mamás como por incluso compañeros varones, trabajadores, nos dijeron que qué bueno que teníamos ese espacio porque luego se daban cuenta que sus compañeras por ahí en las oficinas sufriendo, entraban a una sala de juntas, entonces no tenían ese espacio digno y privado para la extracción de su leche. Entonces creo que ese fue el principal beneficio, que se sintieron en confianza y el ambiente entre las madres trabajadoras fue benéfico…" (*Manager and HR personnel, Guadalajara, [RRHH02])*

[Importance of a BF-friendly environment in the workplace] "...Sé que a ellas les da tranquilidad saber que hay un lugar seguro, tranquilo para usar en caso de que sea necesario, y obviamente, digo, pues la verdad también es un tema muy privado de ellas que tampoco andan platicando con todos, ¿no?, pero por lo menos a mí sí me dijeron "yo sí prefiero hacerlo en mi casa, pero si un día se necesita hacerlo en la oficina, pues es mucho mejor en una sala de lactancia que en el baño o pedir permiso para salir", por lo que sea, por limpieza, por todo. Entonces, la verdad creo que es algo que poco a poco empieza a ser como algo básico a tener…" *(Male employee, Monterrey, [H01])*

[Perception that the number of potential users is limited] "… Mmmm... pues no sé como que... no, no sé. O sea, como que siento que pues ya pasó ese tiempo para mí, estuve bien, no tengo una queja, entonces, también no creo que seamos tantas que tengamos hijos, entonces, si la pregunta es si lo veo necesario, la verdad no. Fuera, por ejemplo, una maquiladora, que pues la mayoría son mujeres a lo mejor sí…" *(Beneficiary or potential beneficiary, Chihuahua, [B03])*

[Perception that flexible work schedules and lactation room usage are mutually exclusive] "… Pues más que voluntario

that mothers do not feel included, in terms of the compatibility of work and family life, I believe that in the sense of belonging to the workplace. It has favored the work environment and moms feel more accepted. The truth is that it was really appreciated both by the mothers and even by male workers. They told us that it was good that we had this space because later they realized that their female colleagues were suffering in the offices, they would go into a meeting room, so they did not have this decent and private space for milk extraction. So I think that was the main benefit, that they felt confident and the environment among the working mothers was favorable…" *(Manager and HR personnel, Guadalajara, [RRHH02])*

[Importance of a BF-friendly environment in the workplace] "...I know that it gives them peace of mind to know that there is a safe, quiet place to use in case it is necessary, and obviously, I mean, the truth is that it is also a very private issue for them and they don't go around talking to everyone, right? But at least I was told "I do prefer to do it at home, but if one day I need to do it at the office, it is much better to do it in a lactation room than in the bathroom or to ask permission to go out", for whatever reason, for cleanliness, for everything. So, the truth is that I think it is something that little by little is starting to be something basic to have…" *(Male employee, Monterrey, [H01])*

[Perception that the number of potential users is limited] "... Mmmm.... well, I don't know how... no, I don't know. I mean, I feel that time has passed for me, I was fine, I don't have a complaint, so, I don't think there are that many of us who have children, so, if the question is whether I think it is necessary, I really don't. Outside, for example, a clothing manufacturing plant, where most of them are women, maybe yes…" *(Beneficiary or potential beneficiary, Chihuahua, [B03])*

[Perception that flexible work schedules and lactation room usage are mutually exclusive] "... Well, more than being

debería de ser una política de la institución, pero siempre en el orden de la necesidad primaria de las mamás que estén lactando en ese tiempo. Si en una institución como la nuestra hay una o dos personas que están lactando y se les pregunta: "¿quieres lactar aquí dentro de tu horario"?, por la comodidad de la mamá diría: "no, prefiero salir más temprano" […] si mañana mismo envío una invitación: "mujeres mamás, no se preocupen, en agosto vamos a tener un lactario, no se preocupen por su horario", me atrevería a responder que las mamás me dirían: "no, no, mejor me voy temprano" [...] yo casi me atrevo a decir que preferirían irse temprano. ¿Por qué?, pues porque para ellas es mucho más cómodo hacer o relajarse de su trabajo e irse a atender a su bebé fuera de aquí…" (*Manager and HR personnel, Chihuahua, [RRHH])*

voluntary, it should be a policy of the institution, but always in the order of the primary need of the mothers who are breastfeeding at that time. If in an institution like ours there are one or two people who are breastfeeding and they are asked "do you want to breastfeed here within your schedule?", for the comfort of the mother she would say: "no, I prefer to leave earlier" [...] if tomorrow I send an invitation: "mothers, don't worry, in August we will have a lactation room, don't worry about your schedule". I would dare to answer that the mothers would tell me: "no, no, I'd better leave early" [...] I almost dare to say that they would prefer to leave early. Why? because for them it is much more comfortable to do or relax from work and go to take care of their baby outside…" *(Manager and HR personnel, Chihuahua, [RRHH])*

[Perception that flexible work schedules and lactation room usage are mutually exclusive] "… Lo que pasa que, cuando la mujer vuelve de incapacidad tiene dos opciones, que es tener los breaks para poder sacarse la leche o puede entrar tarde o salir temprano. Entonces la mayoría de las mujeres de aquí han escogido salir temprano o entrar tarde, entonces a lo mejor por eso no sé había hecho la necesidad grande de tenerlo, pero sin embargo, sabemos como empresa debemos de contar con la sala de lactancia para darle la comodidad a la persona y la higiene que merece…" (*Manager and HR personnel, Chihuahua, [RRHH01])*

[Perception that flexible work schedules and lactation room usage are mutually exclusive] "... What happens is that when the woman returns from disability she has two options, which is to have the breaks to be able to extract her milk or she can come in late or leave early, so most of the women here have chosen to leave early or come in late. So maybe that is why there was no great need to have it (lactation room), but nevertheless, we know as a workplace we must have a lactation room to give the person the comfort and hygiene that she deserves…" *(Manager and HR personnel, Chihuahua, [RRHH01])*

[Benefits beyond the law] "… Se tiene un grupo que lleva la jefa de Relaciones Laborales, que se llama "*Women at XXX*", entonces hay reuniones de temas de liderazgo, pláticas… uno de los temas en algún momento fue el del lactario, el embarazo, igualdad de oportunidades, varios temas que tocaban, pláticas directas con el director, estaba muy padre. Entonces, nosotros tenemos muy claro el tema de fomentar la lactancia materna…" *(Manager and HR personnel, Monterrey, [RRHH01])*

[Benefits beyond the law] "... We have a group led by the head of Labor Relations, called "Women at *XXX*", so there are meetings on leadership issues, talks... one of the topics at some point was the breastfeeding program, pregnancy, equal opportunities, several topics that were discussed, direct talks with the director, it was very cool. So, we are very clear on the issue of promoting breastfeeding…" *(Manager and HR personnel, Monterrey, [RRHH01])*

[Perception that the number of potential users is limited] “... (se le preguntó si considera necesario un lactario) Bueno, si nuestro índice de maternidad fuera más alto creo que sí sería necesario, definitivamente sería un sí, estaríamos pensando en poner un lactario, pero es tan bajo nuestro índice que sería una facilidad, un espacio ocioso que no usaríamos…” *(Manager and HR personnel, Chihuahua, [RRHH01])*

[Perception that the number of potential users is limited] ".... (was asked if a lactation room is necessary) Well, if our maternity rate were higher I think it would be necessary, it would definitely be a yes, we would be thinking about having a lactation room, but our rate is so low that it would be a facility, an useless space that we wouldn't use..." *(Manager and HR personnel, Chihuahua, [RRHH01])*

**Physical environment**	[No physical space] "… Sí, porque todo es de vidrio, entonces era muy complicado porque era un local muy grande donde yo nada más estaba con dos hombres, éramos dos hombres y yo, entonces sí les decía: "muchachos, me voy a encerrar en el clóset, no pasen a la cocina, me voy a estar sacando leche, por si alguien pregunta por mí", no hubo ningún problema. El clóset estaba muy chiquito […] tenía yo ahí mi silla, mi conector, me sacaba la leche, la guardaba en el refrigerador…" *(Beneficiary or potential beneficiary, Chihuahua, [B02])*	[No physical space] "... Yes, because everything is made of glass, so it was very complicated because where I was, it was a very big place, there were two men and I, so I told them: "guys, I'm going to lock myself in the closet, don't go to the kitchen, I'm going to extract milk, in case someone asks for me", there was no problem. The closet was very small [...] I had my chair there, my connector, I extracted my milk, I kept it in the fridge…" *(Beneficiary or potential beneficiary, Chihuahua, [B02])*
	[No physical space] "… Fíjate que como amamanté a mi niña, que acaba de cumplir dos años, cuando vine para acá todavía estaba sacándome leche. Entonces lo que hacía, aquí sí estaba un poquito complicado, me metía a la sala de juntas que está ahí, porque mi escritorio estaba pegado a la ventana, y ahí me sacaba o de plano, si ya no aguantaba, me metía al baño y le cerraba, con el permiso de todas mis compañeras, les decía "si quieren entrar, pues me tocan"…" *(Beneficiary or potential beneficiary, Chihuahua, [B02])*	[No physical space] "... Since I breastfed my baby girl, who just turned two years old, when I came here I was still extracting my milk. So what I did, here it was a little complicated, I would go into the meeting room, because my desk was next to the window, and there I would extract my milk or, if I couldn't hold it, I would go into the bathroom and close it, with the permission of all my coworkers, I would tell them "if you want to come in, then knock on the door"…" *(Beneficiary or potential beneficiary, Chihuahua, [B02])*
	[No physical space] "… No y de cierta forma faltan áreas así de ese tipo porque no... bueno, he visto a una de las chicas que tiene bebé, acaba de tener bebé, y se extrae la leche en el baño, entonces, pues como que no es higiénico. Entonces sí a lo mejor faltaría un espacio donde puedas... o sea, uno como mamá tener la oportunidad de extraer la leche..." *(Beneficiary or potential beneficiary, Chihuahua, [B04])*	[No physical space] "... No, and in a certain way there is a lack of such areas because... well, I have seen one of the girls who has a baby, she just had a baby, and she extracts her milk in the bathroom, so it is not hygienic. So maybe we need a space where you can... I mean, as a mother, you can have the opportunity to express your milk…" *(Beneficiary or potential beneficiary, Chihuahua, [B04])*
	[No physical space] "...sí ha habido mamás que se acercan a preguntar si tenemos un lactario. Y bueno, pues no lo tenemos, pero también tenemos la otra parte de mamás que buscan su espacio porque les duele (los pechos), les molesta no extraerse la leche…" (*Beneficiary or potential beneficiary, Guadalajara, [B01]*)	[No physical space] "...there have been mothers who have come to ask if we have a lactation room, and well, we don't have one, but we also have the other part of moms who are looking for their space because it hurts (their breasts), it bothers them not to express their milk…" *(Beneficiary or potential beneficiary, Guadalajara, [B01])*
	[Available physical space] "… En los últimos años precisamente tuvimos, siento yo, colaboradoras más informadas, con más conciencia, que verdaderamente estaban haciendo ese esfuerzo por continuar con la lactancia materna el mayor tiempo posible [...] entonces teníamos por ahí un espacio que se asignaba para el área de archivo y pues lo fuimos acondicionando. [...] pero luego resulta que teníamos dos o tres mamás que estaban en su periodo de lactancia y pues ese espacio ya tampoco era suficiente. Entonces dijimos "oye, sí es necesario el tener un espacio dedicado y que cumpla con todas esas características que permitan que nuestra gente se	[Available physical space] "... In the last few years we had, I feel, more informed collaborators with more awareness who were really making an effort to continue breastfeeding for as long as possible [...] so we had a space that was assigned for the archive area and we were preparing it [...] but then it turned out that we had two or three mothers who were breastfeeding and that space was not enough. So we said "hey, it is necessary to have a specific space that meets all those characteristics that allow our people to feel comfortable" and somehow we also said "well, those who are pregnant now, if they did not have the

**Supervisor support**	[Inadequate support] "…es que perdí perdí mi incapacidad porque mi parto se adelantó, entonces nada más fue un mes y luego ya empecé ir a trabajar. Se supone que en mi trabajo anterior tenía que salir temprano para la lactancia, pero era muy complicado, no era sencillo porque siempre había trabajo demandante. El jefe era el demandante y luego no había un lugar donde pudieras sacar tu leche y guardarla. O sea no, no tenían esas condiciones…" *(Beneficiary or potential beneficiary, Chihuahua, [B16])*<br><br>[Inadequate support] "...creo que existe todavía un poco de machismo de los líderes, a lo mejor. Creo que es parte de no reconocer esto que es importante para el bienestar de sus empleados. Creo que va por ahí…" (*Male employee, Guadalajara, [H02]*)<br><br>[Inadequate support] "… el apoyo que tienes con tu con tu jefe pues tu lo tienes que pedir [...] o sea todo depende del trato entre tu y el jefe, yo no tuve ningún problema porque pues sí le explicaba y devolvía mis horas, no hubo ningún detalle. Pero sí siento que no hay algo que te ayude y te apoye con el proceso, entonces si él no quiere ¿cómo le haces? ni aunque grites o llores, a menos que te corran o te quieras salir ¿no?, pero eso yo pensaba "y si ella no me diera permiso" ¿cómo le voy a hacer?"..." *(Beneficiary or potential beneficiary, Mérida, [B02])*<br><br>[Inadequate support] "… No, de hecho, ese tema no me lo habían tocado y yo se lo tuve que tocar a mi jefe [...] porque estaba viniendo, ya había regresado de mi incapacidad, y yo duré una semana saliendo tarde. Le dije "creo que por ley tengo una prestación o un beneficio como trabajadora, no sé si es media hora o una hora, en diferentes empresas lo trabajan diferente", y me dice "¿pero no es por ley o sí?", y le dije "Sí, creo que sí es por ley", y le tuve que hacer como que copia de la ley y se lo mandé por WhatsApp para que lo leyera y me dijo "bueno, pues te puedes tomar la media hora"…" *(Beneficiary or potential beneficiary, Mérida, [B07])*	[Inadequate support] "...I lost my maternity leave because I gave birth early, so it was only a month and then I started to go to work. In my previous job I was supposed to leave early to breastfeed, but it was very complicated, it was not easy because there was always demanding work. The boss was demanding and then there was no place where you could extract your milk and store it. So, no, they didn't have those conditions…" *(Beneficiary or potential beneficiary, Chihuahua, [B16])*<br><br>[Inadequate support] "...I think there is still a little bit of male chauvinism from the leaders, maybe. I think it's part of not recognizing that this is important for the well-being of their employees. I think it goes that way…" *(Male employee, Guadalajara, [H02])*<br><br>[Inadequate support] "... the support you have with your boss, you have to ask for it [...] it all depends on the relationship between you and the boss, I didn't have any problem because I explained to him and paid back my hours, there was no problem at all. But I do feel that there is nothing to help you and support you with the process, so if he doesn't want to, how do you do it? not even if you scream or cry, unless you get fired or want to leave, but I thought "and if she doesn't give me permission, how am I going to do it?" *(Beneficiary or potential beneficiary, Merida, [B02])*<br><br>[Inadequate support] "... No, in fact, this subject had not been mentioned to me and I had to mention it to my boss [...] because I was coming, I had already returned from my maternity leave, and I had been going out late for a week. I told him "I think that by law I have a provision or a benefit as a worker, I don't know if it is half an hour or an hour, in different workplaces they work it differently", and he said "but it is not by law or yes?", and I said "Yes, I think it is by law", and I had to make him a copy of the law and I sent it to him by WhatsApp so he could read it and he said "well, you can take the half hour"..." *(Beneficiary or potential beneficiary, Mérida, [B07])*

	[Perceived support] "… De hecho, fíjate que no, no lo vi con Recursos Humanos. Siempre he tenido mucho el apoyo de mi jefa, pero sí fuimos y le comentamos a los de Recursos Humanos y ya dijeron "sí, esto está perfecto", y dieron autorización para los recesos…" *(Beneficiary or potential beneficiary, Chihuahua, [B05])*	[Perceived support] "... In fact, I did not see it with Human Resources. I have always had a lot of support from my boss, we went and told Human Resources and they said "yes, this is perfect", and they gave authorization for the breaks…" *(Beneficiary or potential beneficiary, Chihuahua, [B05])*
	[Inadequate support] "...Pero, por ejemplo, ves que el gobierno federal cambia las administraciones, en la administración de mi niña batallé mucho para que me dieran mi oficio de la lactancia. Tuve que buscar en internet, tuve que preguntar en otras universidades porque la persona de Recursos Humanos me decía, "no, pero aquí dice que es a partir de que el niño nace". Entonces yo le dije: "¿Cómo va a ser a partir de que el niño nace?, aquí dice que es cuando te reincorporas al trabajo, ¿de qué sirve que me quites una hora de trabajo cuando yo estoy incapacitada?"…" *(Beneficiary or potential beneficiary, Chihuahua, [B02])*	[Inadequate support] "...But, for example, you know that the federal government changes administrations, in my daughter's administration I struggled a lot to get my breastfeeding paper. I had to look on the internet, I had to ask in other universities because the person from Human Resources told me, "no, but here it says that it is from the moment the child is born". So I told her: "How can it be from the moment the child is born?, it says here that it is when you return to work, what's the point of taking an hour off work when I am on my maternity leave?..." *(Beneficiary or potential beneficiary, Chihuahua, [B02])*
**Co-worker support**	[Perceived support] "… como ya nos acostumbramos a que es algo normal, pues ya todos acatan la decisión, si alguna compañera está en un centro laboral, se le apoya o se pueden resolver los detalles que hay, antes que ella llegue. En dado caso que ella tenga que salir temprano, pues entre todos tratamos de realizar los trabajos que llegaran a haber en la ausencia de esta persona…" *(Male employee, Mérida, [H03])*	[Perceived support] "... since we have become familiar with the fact that it is something normal, we all accept the decision, if a colleague is at a work center, we support her or we can solve the details before she arrives. In the event that she has to leave early, we all try to do the work that will be done in the absence of this person…" *(Male employee, Merida, [H03])*
	[Perceived support] "… Igual la verdad fueron muy respetuosas, ayuda que sean puras mujeres creo que si hubiera algún hombre hubiera sido diferente [...] había una mujer que también daba pecho y pues como que nos apoyamos un poco…" *(Beneficiary or potential beneficiary, Mérida, [B03])*	[Perceived support] "... The truth is that they were very respectful, it helps that they are all women, I think that if there had been a man it would have been different [...] there was a woman who was also breastfeeding and we kind of supported each other a little bit…" *(Beneficiary or potential beneficiary, Mérida, [B03])*
	[Inadequate support] "… Lo que yo he percibido, pero no hemos sondeado, sí hay gente a quien le molesta que las mujeres se vayan antes porque consideran que se les carga	[Inadequate support] "... What I have perceived, but we have not surveyed, there are people who are bothered by the fact that

	mucho el trabajo, pero sinceramente son un par de comentarios aislados, los demás ni lo mencionan, no creo que siquiera lo tengan en mente…" *(Manager and HR personnel, Chihuahua, [RRHH01])*	women leave earlier because they consider that they are burdened with too much work, but honestly they are a couple of isolated comments, the others do not even mention it, I don't think they even have it in mind…" *(Manager and HR personnel, Chihuahua, [RRHH01])*

[1] Quotations have been translated from Spanish to English as expressed by the participants.
CMO: Context-Mechanism-Outcome; BF: breastfeeding; HR: human resources.
